# External validation of risk prediction models for incident colorectal cancer using UK Biobank

**DOI:** 10.1038/bjc.2017.463

**Published:** 2018-01-30

**Authors:** J A Usher-Smith, A Harshfield, C L Saunders, S J Sharp, J Emery, F M Walter, K Muir, S J Griffin

**Affiliations:** 1 The Primary Care Unit, Department of Public Health and Primary Care, University of Cambridge, Cambridge CB2 0SR, UK; 2 MRC Epidemiology Unit, University of Cambridge, Institute of Metabolic Science, Cambridge CB2 0QQ, UK; 3 Department of General Practice, Centre for Cancer Research, Faculty of Medicine, Dentistry and Health Sciences, The University of Melbourne, Victorian Comprehensive Cancer Centre, Melbourne, VIC 3010, Australia; 4 Institute of Population Health, University of Manchester, Manchester M13 9PL, UK

**Keywords:** colorectal cancer, risk, prediction, model, external validation

## Abstract

**Background:**

This study aimed to compare and externally validate risk scores developed to predict incident colorectal cancer (CRC) that include variables routinely available or easily obtainable via self-completed questionnaire.

**Methods:**

External validation of fourteen risk models from a previous systematic review in 373 112 men and women within the UK Biobank cohort with 5-year follow-up, no prior history of CRC and data for incidence of CRC through linkage to national cancer registries.

**Results:**

There were 1719 (0.46%) cases of incident CRC. The performance of the risk models varied substantially. In men, the QCancer10 model and models by Tao, Driver and Ma all had an area under the receiver operating characteristic curve (AUC) between 0.67 and 0.70. Discrimination was lower in women: the QCancer10, Wells, Tao, Guesmi and Ma models were the best performing with AUCs between 0.63 and 0.66. Assessment of calibration was possible for six models in men and women. All would require country-specific recalibration if estimates of absolute risks were to be given to individuals.

**Conclusions:**

Several risk models based on easily obtainable data have relatively good discrimination in a UK population. Modelling studies are now required to estimate the potential health benefits and cost-effectiveness of implementing stratified risk-based CRC screening.

Colorectal cancer (CRC) is the second leading cause of cancer-related death in Europe and the United States (Stewart and Kleihues, 2003). Survival is strongly related to stage at diagnosis (Cancer Research UK, 2009). There is good evidence that screening adults in the general population who are at average risk using faecal occult blood testing (FOBt), flexible sigmoidoscopy or colonoscopy reduces CRC incidence and mortality (Hardcastle *et al*, 1996; Kronborg *et al*, 1996; Lindholm *et al*, 2008; Holme *et al*, 2013; Lin *et al*, 2016). As a result, CRC screening for individuals above a defined age has been introduced in most countries with a high CRC incidence (Schreuders *et al*, 2015). For example, currently in the US, the US Preventive Services Task Force recommends all men and women are invited for screening at age 50 years (US Preventive Services Task Force *et al*, 2016) and in England all men and women aged 60 to 74 are offered FOBt every 2 years (Public Health England, 2015).

However, as with all screening programmes, CRC screening has the potential to cause harm, both directly to those screened and indirectly through diversion of resources away from other services. Targeted or stratified screening could potentially provide a way of reducing complication rates and demand on services by better identifying those who are more likely to benefit from screening and early intervention and potentially inviting them earlier or more frequently.

Such an approach requires risk prediction models capable of stratifying the population. We have previously published a systematic review of risk prediction models for CRC and identified 40 models that could potentially be used for this purpose (Usher-Smith *et al*, 2015). They range from models including only data routinely available from electronic health records such as age, gender and body mass index (BMI), to more complex models containing detailed information about lifestyle factors and genetic biomarkers. Including models published since that review, of the 26 that include variables routinely available or easily obtainable via self-completed questionnaire, where reported (*n*=12), half the models have acceptable-to-good discriminatory ability (C-statistic >0.7) in the derivation sample. However, only five have been validated in external populations (C-statistic 0.60–0.71) and none in a UK population.

UK Biobank is the largest population-based cohort in the UK (Allen *et al*, 2012). In order to inform future risk stratified screening approaches in the UK, we aimed to assess the performance of risk scores that have been developed to identify individuals at higher risk of developing CRC and include only variables routinely available or easily obtainable via self-completed questionnaire, in the UK Biobank cohort.

## Materials and methods

We performed an external validation of risk models following the TRIPOD (transparent reporting of a multivariable prediction model for individual prognosis or diagnosis) guideline (Collins *et al*, 2015).

### Selection of risk prediction models

We identified 40 risk prediction models for either CRC, colon cancer or rectal cancer from our recent systematic review and two that had been published since the end of the search period for that review (March 2014) and November 2016. If insufficient data were provided to operationalise the risk scores in the published articles, we contacted authors asking for the additional data. We excluded 16 that included either biochemical or genetic biomarkers. In three, it was not possible to operationalise the risk score, either because details of how the co-variates were incorporated in the final model were not provided (Bener *et al*, 2010), the model developed was a decision tree (Camp and Slattery, 2002), or the model required data on risk factors over 20 years prior to baseline (Wei EK *et al*, 2009). A further two (Almurshed, 2009; Taylor *et al*, 2011) included risk markers for which there is no comparable variable available within the UK Biobank (region in Riyadh, Saudi Arabia and knowledge of a high-fibre diet, and second and third degree family history, respectively) (Supplementary Figure 1). As Ma *et al* (2010), Freedman *et al* (2009) and Driver *et al* (2007) developed separate models for CRC, colon and rectal cancer on the same data set, we included only the models for CRC. This meant we included 14 risk models in our analysis, 13 with CRC as the outcome and 1 with colon cancer as the outcome (Colditz *et al*, 2000). Details of these models, including the study design, method used to develop them and the risk factors included in each are given in Table 1. Except for the models by Colditz *et al* (2000), Johnson *et al* (2013) and Wei Y-S *et al* (2009), age was included in all the models and alcohol, BMI, smoking and family history were each included in over half. Only one model included sex (Tao *et al*, 2014), whereas six were developed to be applicable to men (Wells *et al*, 2014; Driver *et al*, 2007; Freedman *et al*, 2009; Ma *et al*, 2010; Hippisley-Cox and Coupland, 2015) and three to women (Wells *et al*, 2014; Freedman *et al*, 2009; Hippisley-Cox and Coupland, 2015). Details of the full equations for the risk models are given in Supplementary Table 1.

### Validation cohort

UK Biobank is the largest population-based cohort in the UK with over 500 000 people recruited during 2006–2011. Details of recruitment and data collection are provided in detail elsewhere (Allen *et al*, 2012). In brief, all people aged 40–69 years who were registered with the National Health Service and lived within ∼25 miles of one of the 22 study assessment centres across the UK were invited to participate. From 9.2 million invitations, 503 325 were recruited (5.5%) and attended an assessment centre at which baseline data was collected on their lifestyle, environment, medical history and body composition using touchscreen questionnaires, interviews and physical measurements. The cohort is representative of the UK general population with respect to age, sex, ethnicity and deprivation within the age range recruited, it is however not representative with respect to a variety of sociodemographic, physical, lifestyle and health-related characteristics, with evidence of a ‘healthy volunteer’ selection bias (Fry *et al*, 2017). Compared with the population of England in 2012 from the Office of National Statistics, incidence rates for CRC per 100 000 person-years were lower for all ages between 45 and 74 years except for 50–54 years (Fry *et al*, 2017). For example, at age 60–64, incidence rates in men and women in UK Biobank were 141.2 and 84, respectively, compared with 159.9 and 92.5 in the general population.

Data on cancer incidence up to 30 September 2014 is available for each participant through linkage to national cancer registries. We excluded from the analysis participants with a diagnosis of CRC (ICD9 153.0–153.9, 154.0, 154.1 and 154.8 and ICD10 C18.0–C18.9, C19, C20 and C21.8) prior to recruitment. Of the 502 633 participants within the UK Biobank cohort, 2331 had a prior diagnosis of CRC, three had a date of death prior to baseline recorded and 127 187 did not have follow-up for 5 years. We therefore included 373 112 participants in our primary analysis. Among those there were 1719 (0.46%) cases of incident CRC.

### Risk factor and outcome variables

For each risk factor, we used data collected at the baseline assessment at cohort entry. Full details of the definition of each risk factor and how we operationalised them in the UK Biobank data set and handled missing data are given in Supplementary Table 2. In all cases, we matched variables from the Biobank data set as closely as possible to those described in each model and if there was not an exact match we derived proxy variables. In most cases, we were able to do this by combining existing variables. For some, this was simple, for example, summing beef, pork and lamb consumption to derive a variable for red meat. In some, however, it was more complex and required a number of assumptions. In other cases, where an exact variable did not exist in the Biobank cohort, we derived variables from similar questions. For example, no data are available in Biobank for historic use of aspirin or non-steroidal anti-inflammatory drugs (NSAIDs). We therefore used responses to the question ‘Do you regularly take any of the following? Aspirin, ibuprofen, paracetamol, codeine’ or the presence of a code indicating NSAID use in the list of current regular treatments to categorise individuals as regular or current users and used the mean duration of use from the literature (Hoffmeister *et al*, 2007) to estimate duration of use.

The outcome for each risk model was newly diagnosed CRC using the data from linked cancer registries (ICD10 C18.0–C18.9, C19, C20 and C21.8).

### Data analysis

For all prediction models, we first computed the predicted probability for each participant at baseline. We then assessed the discrimination and calibration of the risk scores. Although some risk models had been developed in all male populations, we assessed the performance in both men and women. Except for the Freedman models (Freedman *et al*, 2009) where the Gauss program available to calculate the risk scores prevents calculation of risk for those outside the defined age range (50–89 years), we assessed the performance of all the models over the full range of UK Biobank participants.

For our primary analysis, we used a ‘complete-case’ approach, including only those for whom a risk score based on all risk factors could be computed and who had 5-year follow-up. This was done on an individual risk score basis so the sample size varies between scores. To reflect the clinical application of risk scores, we did not exclude those who did not have 5-year follow-up due to death. We treated the outcome as a binary variable (developed CRC or did not develop CRC) and compared the overall discriminative ability of the models numerically with the area under the receiver operating characteristic curve (AUC). We also calculated sensitivity, specificity, positive- and negative-likelihood ratios (LR+ and LR−) and the positive and negative predictive values (PPV and NPV) using a cutoff value for each risk score chosen such that 10% of the population had values above the cutoff; the procedure was then repeated using cutoffs where 20, 80 and 90% had values above the cutoff.

If data were available in the original published reports or from authors, we assessed calibration graphically by comparing the predicted risk with the observed percentage of those who developed CRC over the 5-year follow-up period stratified by deciles and calculated Hosmer–Lemeshow statistics. QCancer10 was the only model to provide data on 5-year risk. All the other models predicted risk over 10 or 20 years and this required converting the predicted risks to risks over 5 years. We did this first assuming a constant risk over time as the rate of incident CRC observed within the UK Biobank cohort was constant over the follow-up period. We then repeated the analysis assuming risk doubles every 5 years, in line with reported increasing incidence rates with increasing age (Cancer Research UK, 2017). To allow comparison across all the models, we also used this same approach for the QCancer10 model.

We then carried out a number of sensitivity analyses. In the first set, we explored the impact of missing data, comparing the performance of the models using the complete-case analysis with an extreme case in which risk factors with >5% missing data were coded as the 90th or 10th percentile values for continuous variables and present or absent for dichotomous. Second, in view of the absence of data on historic aspirin or NSAID use and inability to distinguish between oestrogen-containing contraceptive pills and progesterone-only pills, we assessed the performance of the models excluding variables for aspirin, NSAIDs or hormonal medication. Third, recognising that these models may be used in multiple countries, we assessed the performance of the QCancer10 model for men without the term for deprivation. As participants with previous colorectal polyps or a diagnosis of inflammatory bowel disease (IBD) would likely be in surveillance programmes, we also assessed the discrimination after excluding those individuals with a history of a colorectal polyp or diagnosis of IBD at baseline. Finally, we compared the performance of the risk scores using an open cohort design, that is to say including participants with <5 years follow-up. In that analysis, we used Harrell’s C-statistic to assess discrimination as it accounts for censoring in survival models (Chambless and Diao, 2006).

All analyses were carried out in Stata 13.1 (StataCorp, 2013).

## Results

The characteristics of the study population are shown in Table 2. Compared to those who did not develop CRC, those who did were on average older and more likely to be male, report a family history of CRC, be a former smoker, eat red meat ⩾3 times per week, use NSAIDs or aspirin currently and have a higher BMI. There was <5% missing data for all the risk factors included in the models with the exception of physical activity for which data were missing for 12% of participants.

### Discrimination

**Figure 1:**
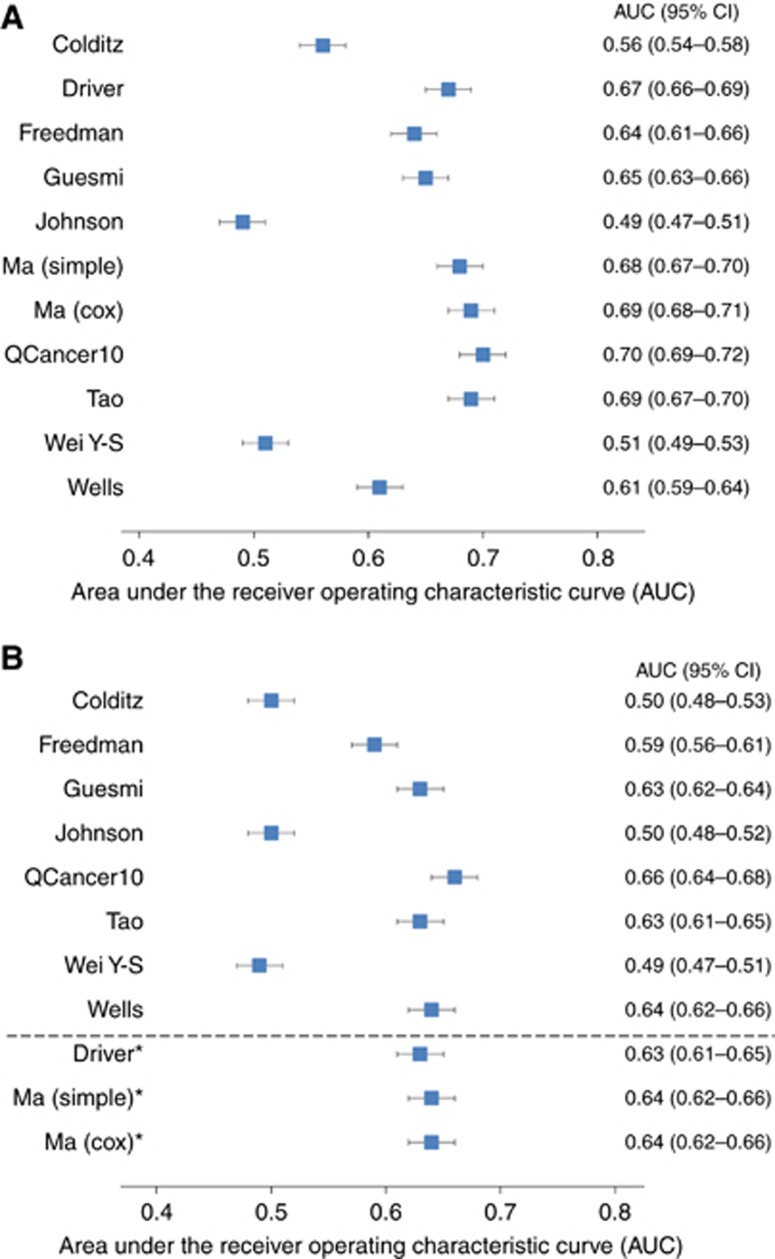
**Model discrimination.** Area under the receiver operating characteristic curve for the risk models in (**A**) men and (**B**) women. *Models originally only developed in men.

Figures 1A and B show the AUC for the 10 models in men and women, respectively. The three models by Colditz *et al* (2000), Johnson *et al* (2013) and Wei Y-S *et al* (2009) that do not include age have the poorest discrimination with all having AUCs <0.6. In men, the QCancer10 model (Hippisley-Cox and Coupland, 2015) and models by Tao *et al* (2014), Driver *et al* (2007) and Ma *et al* (2010) all had AUCs over 0.67. In general, the discrimination was less good in women. Of the models developed for women, the QCancer10 (Hippisley-Cox and Coupland, 2015), Tao *et al* (2014), Guesmi *et al* (2010) and Wells *et al* (2014) models were the best performing with AUCs between 0.63 and 0.66. When applied to women, the Driver *et al*, (2007) and Ma *et al* (2010) models had AUCs of 0.63 (95% CI: 0.61–0.65) and 0.64 (95% CI: 0.62–0.66), respectively.

The sensitivity, likelihood ratios and PPV and NPVs are also shown in Tables 3 and 4. By targeting the 10% with the highest risk, the QCancer10 (Hippisley-Cox and Coupland, 2015), Ma *et al* (2010) and Wells *et al* (2014) models identified between 24% and 26% of men and 19% and 20% of women who went on to develop CRC. In women the Johnson model (Johnson *et al*, 2013) also had a sensitivity of 19.8 for the top 10%. This compares to 17 and 16% for the UK screening programme age threshold for men and women, respectively. Among those with the highest 20% risk, this increased to 37–43% for men and 33–36% for women, compared with 31% for the UK screening programme age threshold. The Driver model (Driver *et al*, 2007), which includes only age, BMI, smoking status and whether individuals consume alcohol, identified 20.2% of men and 17.4% of women who went on to develop CRC by targeting the 10% at highest risk and 38.5% of men and 30.9% of women by targeting the 20% at highest risk. The NPVs were high and comparable (>99.4) for all models.

### Calibration

**Figure 2:**
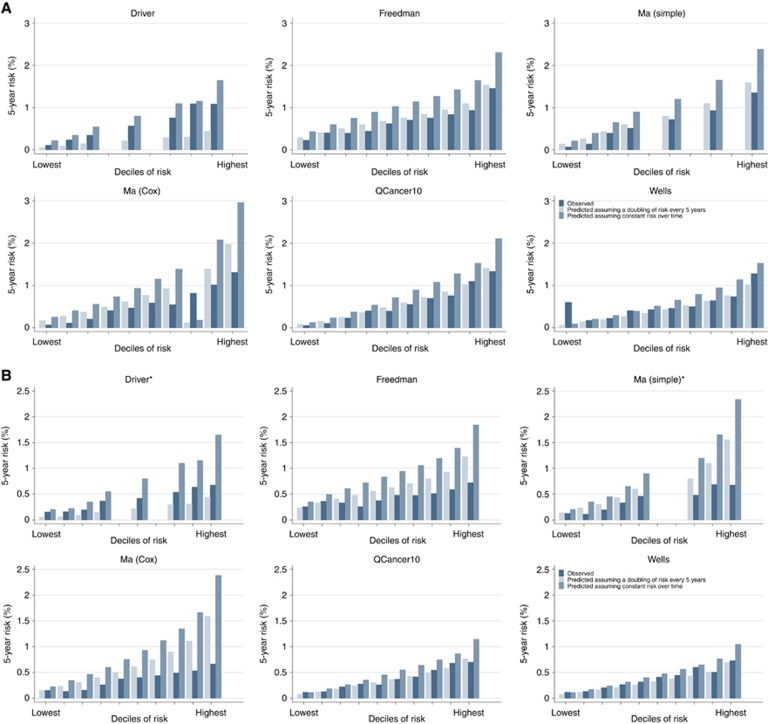
**Model calibration.** Plots of observed and predicted 5-year risk of colorectal cancer for (**A**) men and (**B**) women. *Models originally only developed in men.

Assessment of calibration was only possible for six of the models Driver *et al*, 2007; Freedman *et al*, 2009; Ma *et al*, 2010; simple; Ma *et al*, 2010 (Cox); QCancer10 (Hippisley-Cox and Coupland, 2015) and Wells *et al*, 2014. Figures 2A and B show the observed and predicted risks of CRC for those models for men and women. When the risk of CRC over time was assumed to be constant all overestimated risk, particularly at higher deciles of risk and in the models developed in men when applied to women (Hosmer–Lemeshow *P*<0.0001 for all risk models). The two Ma models and Freedman model also overestimated risk in both men and women when the risk was assumed to double every 5 years, while the Driver model, which was the only model initially developed to estimate risk over a 20-year period, underestimated risk. The predicted risks from the QCancer10 and Wells models more closely matched the observed risks when the risk was assumed to double every 5 years, although overall calibration remained poor (Hosmer–Lemeshow *P*<0.05). When using the published algorithm for 5-year risk for the QCancer10 models, both the male and female models also overestimated risk (Hosmer–Lemeshow *P*<0.05) (Supplementary Figure 2).

### Sensitivity analyses

The results from all the sensitivity analyses (Supplementary Tables 3 and 4) were consistent with the main analysis: the confidence intervals for the AUC and the C-statistic in the open cohort analysis (Supplementary Table 5) for each model overlapped the AUC obtained in the main closed-cohort analysis. As the Colditz model (Colditz *et al*, 2000) was developed to predict colon cancer rather than CRC, we also assessed the discrimination with colon cancer as the outcome. The AUCs for that analysis were also within the confidence interval of those with CRC as the outcome (men AUC 0.57 (95% CI 0.55–0.59); women AUC 0.51 (95% CI 0.49–0.54)).

## Discussion

### Principal findings

In this large, UK population-based study, we found that the performance of published risk models for CRC varied substantially. Of the 14 risk models for CRC identified from an update of an existing systematic review (Usher-Smith *et al*, 2015), the QCancer10 model (Hippisley-Cox and Coupland, 2015) and models by Tao *et al* (2014), Driver *et al* (2007) and Ma *et al* (2010) had the highest discrimination in men with all having AUCs over 0.67. Discrimination was lower for women: the QCancer10 (Hippisley-Cox and Coupland, 2015), Ma *et al* (2010) and Wells *et al* (2014) models were the best performing with AUCs between 0.64 and 0.66. The risk models that do not include age Colditz *et al* (2000); Johnson *et al* (2013) and Wei Y-S *et al* (2009) performed least well. The QCancer10 model (Hippisley-Cox and Coupland, 2015), Tao *et al* (2014) and Ma *et al* (2010) had the highest sensitivities in men. For each of these models, the top 10% included between 24% and 26% of those who went on to develop CRC, and the top 20% between 41% and 43%. For women, the models by Wells *et al* (2014), Ma *et al* (2010), and QCancer10 (Hippisley-Cox and Coupland, 2015) also had the highest sensitivities, alongside the model by Johnson *et al* (2013). Calibration was sensitive to assumptions about the change in risk over time, with all models overestimating risk when risk was assumed to be constant over time and estimated risks more closely matching observed risk when risk for each individual was assumed to double every 5 years.

The finding that the three poorest performing risk models in both men and women Colditz *et al* (2000),Johnson *et al* (2013); Wei Y-S *et al* (2009) are the ones that do not include age, and of those only the Colditz model in men performs better than chance, highlights the importance of older age as a risk factor for development of cancer. We also found that discrimination is poorer in women than in men for all except the Wells model (Wells *et al*, 2014). This may relate to a difference in the reporting of risk factors or a difference in the aetiology of the disease between sexes. For example, it is known that a higher proportion of women present with right-sided colon cancer than men (Hansen and Jess, 2012). The molecular and pathological characteristics of CRC differ depending on tumour location and studies have reported different associations between dietary factors (Kim *et al*, 2015) and CRC risk by sex. The impact of female hormonal factors may also be complex, with previous and current hormone replacement therapy associated with a decreased risk, while chronic endogenous oestrogen exposure may be associated with an increased risk in postmenopausal women (Lin *et al*, 2012; Bae *et al*, 2013).

The finding that the only risk model included which was developed in a UK population, QCancer10, had the highest discrimination in this UK cohort also suggests that the distribution and impact of risk factors may differ geographically. Country-specific risk models may therefore be preferable when implementing stratified screening programmes.

### Strengths and limitations

To our knowledge, this is the first study to directly compare multiple published risk prediction models for CRC in the same population, and the first to externally validate any risk prediction models in a UK population. By identifying models for inclusion from an update of an existing systematic review (Usher-Smith *et al*, 2015) and contacting authors concerning missing data, we have been able to include 14 risk models developed around the world. There were, however, six identified models that we were unable to validate: four where it was not possible to operationalise the risk score and two where variables were not present in the UK Biobank cohort. We think it is unlikely that the models by Almurshed, Taylor or Benner (Almurshed, 2009; Bener *et al*, 2010; Taylor *et al*, 2011) would perform better than those included as they do not include age and neither of the models by Camp and Slattery (2002) or Wei EK *et al*, (2009) have been externally validated, but both had only moderate discrimination in development populations (AUC 0.61). Advantages of using the UK Biobank cohort include the large size, comprehensive phenotyping, completeness of data and linkage to national cancer registries. However, the response rate to invitations to take part was only 5.5% (Allen *et al*, 2012). While the cohort is representative of the UK general population with respect to age, sex, ethnicity and deprivation within the age range recruited, it is however not representative with respect to a variety of sociodemographic, physical, lifestyle and health-related characteristics (Fry *et al*, 2017). For example, mean BMI in the UK Biobank men and women aged 55–64 years was 27.9 and 27.3, respectively, compared with 28.5 and 28.0 in the general population who took part in the Health Survey for England 2008, UK Biobank men and women were less likely to be current smokers than the general population and incidence rates of CRC were lower in the UK Biobank population. Although representative population samples may not always be necessary to make generalisable conclusions about associations between exposures and disease (Collins, 2012), the performance of risk prediction models should ideally be assessed within the population in which they are going to be used (Collins *et al*, 2015). The performance of the risk models in this study may, therefore, not reflect those in the entire UK population or other populations and the ‘healthy volunteer’ selection bias may partly explain the finding that some of the models overestimated absolute risk. This ‘healthy volunteer’ bias will have less influence over the relative risk, and hence discrimination of the models. Nevertheless, the average population risk may be lower in the UK Biobank than across the whole UK population and the discrimination likely underestimated due to a narrower range of risk. The relatively short duration of follow-up to date within UK Biobank also means that we were only able to evaluate calibration with estimates of risk over a 5-year period. To do this required us to make assumptions about the pattern of CRC risk over time. While this increases the uncertainty for each model, by choosing to present data for the situation in which risk is constant over time, as in our data, and one in which it doubles every 5 years, we provide the range of likely values. We were also only able to do this for models in which it was possible to calculate an estimated absolute risk from the original publication. It is also not possible from the data to distinguish between those individuals diagnosed with incident CRC through surveillance and those diagnosed following symptoms. Although the quality of the evidence is low, a recent Cochrane review showed that colonoscopic surveillance in patients with IBD may reduce the development of CRC and the rate of CRC-associated death through early detection (Bye *et al*, 2017). The incidence of CRC over the 5-year period used in this study may therefore be either higher or lower. Our sensitivity analysis excluding individuals likely to be in a surveillance programme (those with a previous polyp or diagnosis of IBD), however, showed no difference in the discrimination of the models.

We also excluded two models identified from our systematic review because they included variables not present in UK Biobank, and had to derive proxy variables if there were no exact matches for many of the risk models. In most cases, we were able to do this by simply combining existing variables, but some, notably aspirin/NSAID use and oestrogen use, required a number of assumptions that may have reduced AUC values. For example, the absence of data in UK Biobank on long-term use of aspirin/NSAIDs meant that we relied on responses to questions about use of aspirin or ibuprofen and the current medication lists to identify current users and were unable to identify past users. In doing this, we may have overestimated those regularly taking aspirin/NSAIDs and are unable to distinguish between those taking aspirin/NSAIDs at high doses for short-term pain relief or low doses for long-term prevention of cardiovascular diseases. This may explain the finding that a greater proportion of those who developed CRC were coded within our data as current users compared to those who did not develop CRC, which is counter to evidence from aetiological and mechanistic studies (Rigas and Tsioulias, 2015; Burn and Sheth, 2016). We think it is unlikely that this is due to reverse causality as it is not current practice to recommend aspirin or NSAIDs to those at high risk of CRC. Instead, as the population who develop CRC are older and conditions requiring medication for pain relief are more common with age, this may be explained by confounding. As a consequence of this, the contribution of aspirin/NSAID use in the five models (Colditz *et al*, 2000; Freedman *et al*, 2009; Tao *et al*, 2014; Wells *et al*, 2014; Johnson *et al* 2013) that include that variable will be reduced and the discrimination potentially underestimated. This is further supported by our sensitivity analysis in which removing the terms for aspirin/NSAID use did not affect the AUC. The models that did not include any variables for which assumptions had been made were those by Driver, Ma, Wei Y-S and the QCancer10 models. This may in part explain why the Driver, Ma and QCancer10 models performed better than many others in this analysis, particularly in men. The limitations of using the AUC to compare across the risk models must also be appreciated. While the AUC is widely considered the standard measure of discrimination and summarises the model performance over all possible thresholds, it does not distinguish between false-positive and false-negative misclassification and is independent of prevalence (Lobo *et al*, 2007). Other approaches, including net reclassification, have been developed to account for these limitations but they are more relevant for detailed comparison of two nested models, rather than for a general comparison of 14 non-nested models. For these reasons, we presented the sensitivity, specificity, PPV and NPV at four thresholds to provide additional comparative information about the potential clinical utility of the models.

### Comparison with existing literature

Overall, the discrimination of the best performing models is a little less good than risk models for other cancers, such as breast (0.72–0.76) (Amir *et al*, 2010) and melanoma (0.70–0.79) (Usher-Smith *et al*, 2014). Our AUC results are similar to those reported in validation studies for the Driver, Freedman, Ma, Tao and Wells models: 0.67 (95% CI: 0.66–0.69) compared with 0.69 for the Driver model in men (Driver *et al*, 2007); 0.64 (95% CI: 0.61–0.66) for men and 0.59 (95% CI: 0.56–0.61) for women compared with 0.61 (95% CI, 0.60–0.62) for men and 0.61 (95% CI, 0.59–0.62) for women for the Freedman model in an external population in America (Park *et al*, 2009); 0.69 (95% CI: 0.68–0.71) compared with 0.64 (0.61–0.67) for the Ma model in an external population in Japan (Ma *et al*, 2010); 0.69 (0.67–0.70) compared with 0.68 (0.57–0.79) in an external population in Germany for the Tao model (Tao *et al*, 2014); and 0.64 (0.62–0.66) in women and 0.61 (0.59–0.64) in men compared with 0.68 (0.67–0.69) in both men and women in 10-fold cross validation of the Wells model (Wells *et al*, 2014). There are no published data on performance in either development populations or validation cohorts for the models by Guesmi *et al* (2010), Johnson *et al* (2013) and Wei Y-S *et al* (2009).

Despite having the highest discrimination in this study population, the AUC for both male and female QCancer10 models were lower than in split-sample validation (Hippisley-Cox and Coupland, 2015) (0.66 (0.64–0.67) and 0.70 (0.69–0.72) compared with 0.85 (0.84–0.85) and 0.86 (0.86–0.87) for women and men, respectively). As for all risk models, this difference may reflect a difference in the incidence of disease or underlying distribution of risk factors within the development and validation populations, or a difference in the collection and/or coding of those underlying risk factors.

For the Colditz model (Colditz *et al*, 2000), we were also only able to assess the discrimination of the point score component of the risk model as the data on population average risk of cancer and cumulative age- and sex-specific 10-year risk incidence used to estimate an individual-based relative risk in the model are not published. This may explain why the discrimination in this study (0.56 (0.54–0.58) in men and 0.50 (0.48–0.53) in women) is lower than in a previous external validation studies (0.71 (0.68–0.74) in men and 0.67 (0.64–0.70) in women) (Kim *et al*, 2004).

### Implications for clinicians and policymakers

This study shows that the performance of published risk models varies substantially with several risk models based on easily obtainable data, such as age, sex, BMI, smoking, alcohol consumption and physical activity, having relatively good discrimination in a UK population. Using the QCancer10 (Hippisley-Cox and Coupland, 2015) model, for example, the data from this study estimates that the top 10% would include 25% of men who later go on to develop CRC, and the top 20% would include 43%. The QCancer10 model includes variables available within routine electronic health records and so would not require additional data collection if access to those records could be used to identify those eligible for screening. Excluding the term for deprivation in the male model also made little difference to the discrimination, so could be removed for use in countries outside the UK. The model by Driver *et al* (2007) also contains variables that would be available within routine health records or easily obtainable (age, BMI, smoking status, alcohol consumption). The discrimination and sensitivity are slightly lower than QCancer10, but the advantage would be simplified data collection or extraction and this may be preferable particularly in health systems where less data are routinely collected.

### Unanswered questions and future research

While this study can help guide the choice of risk prediction model to identify those at higher risk of CRC, these findings do not tell us the extent to which using these models in place of the current age-based criteria might improve efficiency or allow us to make recommendations about different tests, screening intervals, preventive advice, treatment, or age of onset of screening based on modelled risk. To answer those questions, modelling studies are needed to explore the potential health benefits and cost-effectiveness of different strategies. These could be performed using microsimulation models, such as the SimCRC (Loeve *et al*, 1999) or MISCAN-COLON (Loeve *et al*, 1999), which simulate the development of adenomas and their progression to CRC in a large population of individuals with distributions of risk factors reflecting those found in the general population. By calculating baseline risks for the simulated population using the best performing models and then modelling age of onset of screening and choice of test using a range of thresholds based on estimated risk, it would be possible to estimate the expected number of CRC deaths prevented, the quality of life years gained and the cost-effectiveness of the screening programme compared to current practice. Implementation studies, ideally randomised controlled trials, are then needed to assess the feasibility of obtaining the risk factor data for each individual, the acceptability of incorporating a stratified approach and potential benefits and adverse consequences of incorporating such an approach into practice. We have also only included risk models based on phenotypic, medical history and lifestyle information in this study as such variables are either routinely available or easily obtainable via self-completed questionnaire. A number of risk models incorporating both genetic and non-genetic biomarkers also exist and may have improved discrimination and calibration. While introducing these into current practice would require fundamental changes in infrastructure (Aronson and Rehm, 2015), progress in this area is advancing (Hayward *et al*, 2017), and simple risk models, such as those in this study, might be useful to identify those in whom collection of additional biomarker information might be helpful. Further research is therefore needed to assess the performance of models incorporating these additional variables.

## Figures and Tables

**Table 1:** Details of the development and factors included in each of the risk scores included in validation study

					**Factors included in score**										
**Author, year**	**Country**	**Cancer outcome**	**Study design**	**Model type**	**Age**	**Sex**	**Ethnicity**	**BMI**	**FH**	**Smoking**	**Alcohol**	**Physical activity**	**Red meat**	**Aspirin/NSAIDs**	**Other**
Colditz *et al*, 2000	USA	Colon	Expert consensus	Expert consensus				•	•		•	•	•	•	Vegetables, height, HRT, birth control pills. multivitamin use, IBD, saturated fat, calcium and vitamin D supplements
Driver *et al*, 2007	USA	Colorectal	Cohort	Logistic regression	•	A		•		•	•				
Freedman *et al*, 2009	USA	Colorectal (male)	Case–control	Logistic regression	•	B	•	•	•	•		•		•	Vegetables, previous sigmoidoscopy or colonoscopy, previous polyps
Freedman *et al*, 2009	USA	Colorectal (female)	Case–control	Logistic regression	•	B	•	•	•			•		•	Vegetables, previous sigmoidoscopy or colonoscopy, previous polyps, oestrogen use
Guesmi *et al*, 2010	Tunisia	Colorectal	Case–control	Logistic regression	•										Processed meat, milk
Johnson *et al*, 2013	Worldwide	Colorectal	Meta-analysis	Meta-analysis				•	•	•	•	•	•	•	IBD, hormone therapy, processed meat
Ma *et al*, 2010	Japan	Colorectal	Cohort	Cox regression	•	A		•		•	•	•			
Ma *et al*, 2010	Japan	Colorectal	Cohort	Simple score	•	A		•		•	•	•			
Tao *et al*, 2014	Germany	Colorectal	Cross-sectional	Logistic regression	•	•			•	•	•		•	•	Previous polyp, previous colonoscopy
Wei Y-S *et al*, 2009	China	Colorectal	Case–control	Logistic regression				•	•	•	•				
Hippisley-Cox and Coupland, 2015 (QCancer10)	UK	Colorectal (male)	Cohort	Cox regression	•	B	•	•	•	•	•				Deprivation, blood cancer, ulcerative colitis, lung cancer, oral cancer, polyps, diabetes
Hippisley-Cox and Coupland, 2015 (QCancer10)	UK	Colorectal (female)	Cohort	Cox regression	•	B	•		•	•	•				Breast cancer, cervical cancer, ulcerative colitis, ovarian cancer, polyps, diabetes, uterine cancer
Wells *et al*, 2014	USA	Colorectal (male)	Cohort	Cox regression	•	B	•	•	•	•	•	•	•	•	Years of education, multivitamins, diabetes
Wells *et al*, 2014	USA	Colorectal (female)	Cohort	Cox regression	•	B	•	•	•	•	•			•	Years of education, multivitamins, diabetes, oestrogen

Abbreviations: A=model applicable to male only; B=different models for male and female; BMI=body mass index; FH=family history; IBD=inflammatory bowel disease; NSAIDs=non-steroidal anti-inflammatory drugs.

**Table 2:** Characteristics of participants in the UK Biobank cohort with at least 5 years follow-up used for validation, including distribution of variables between those with and without incident colorectal cancer

	**No incident CRC,** ***n*****=371 393**	**Incident CRC,** ***n*****=1719**	**% with incident CRC**
**Age (years)**			
Mean (SD)	56.4 (8.1)	61.3 (6.3)	—
Missing (%)	0 (0)	0 (0)	—
**Sex**			
Male (%)	168 757 (45.4)	965 (56.1)	0.57
Female (%)	202 636 (54.6)	754 (43.9)	0.37
Missing (%)	0 (0)	0 (0)	—
**Ethnicity**			
White (%)	354 928 (95.6)	1 668 (97.0)	0.47
Other (%)	14 787 (4.0)	43 (2.5)	0.29
Missing (%)	1678 (0.5)	8 (0.5)	0.47
**Years of full-time education**			
Mean (SD)	13.0 (2.8)	12.7 (2.8)	—
Missing (%)	7677 (2.1)	34 (2.0)	—
**BMI (kg/m^2^)**			
<20 (%)	8631 (2.3)	31 (1.8)	0.35
20–24.9 (%)	113 797 (30.6)	449 (26.3)	0.39
25–29.9 (%)	157 093 (42.3)	778 (45.6)	0.50
≥30 (%)	90 008 (24.2)	449 (26.3)	0.50
Missing (%)	1864 (0.5)	12 (0.7)	0.64
**Family history of CRC^a^**			
Yes (%)	40 131 (10.8)	249 (15.2)	0.62
No (%)	316 589 (85.2)	1392 (84.8)	0.44
Missing (%)	14 673 (4.0)	78 (4.5)	0.53
**Smoking status**			
Never (%)	201 821 (54.3)	760 (44.6)	0.38
Former (%)	127 793 (34.4)	767 (45.0)	0.60
Current (%)	39 864 (10.7)	177 (10.4)	0.44
Missing (%)	1915 (0.5)	15 (0.9)	0.78
**Alcohol drinking status**			
Non (%)	15 730 (4.2)	59 (3.4)	0.37
Former (%)	13 194 (3.6)	66 (3.9)	0.50
Current (%)	341 591 (92.0)	1589 (92.7)	0.47
Missing (%)	878 (0.2)	5 (0.3)	0.57
Physical activity (MET-h/d)			—
Mean (SD)	28.3 (5.5)	28.6 (5.8)	—
Missing (%)	43 461 (11.7)	235 (13.7)	
**Red meat consumption**			
<3 times/week (%)	283 455 (76.3)	1244 (72.4)	0.44
≥3 times/week (%)	82 959 (22.3)	452 (26.3)	0.54
Missing (%)	4979 (1.3)	23 (1.3)	0.46
**Current aspirin use**			
Yes (%)	55 702 (15.0)	347 (20.5)	0.62
No (%)	311 590 (83.9)	1348 (79.5)	0.43
Missing (%)	4101 (1.1)	24 (1.4)	0.58
**Current NSAID use**			
Yes (%)	116 057 (31.3)	568 (33.5))	0.49
No (%)	251 5775 (67.7)	1129 (66.5)	0.45
Missing (%)	3759 (1.0)	22 (1.3)	0.58
**Fruit and vegetable consumption**			
<5 portions/day (%)	82 361 (22.2)	389 (22.6)	0.47
≥5 portions/day (%)	280 637 (75.6)	1290 (75.0)	0.46
Missing (%)	8395 (2.3)	40 (2.3)	0.48

Abbreviations: BMI=body mass index; CRC=colorectal cancer; MET-h/d=Metabolic Equivalent of Task hours per day; NSAID=non-steroidal anti-inflammatory drug.

a One of more of mother, father or sibling.

**Table 3:** Discriminatory performance measures for each of the risk models for 5-year risk of developing colorectal cancer in men

	**Colditz**	**Driver**	**Freedman**	**Guesmi**	**Johnson**	**Ma (simple)**	**Ma (Cox)**	**QCancer10**	**Tao**	**Wei Y-S**	**Wells**
**Total n**	***n*****=139 257**	***n*****=167 762**	***n=*****101 530**	***n*****=168 825**	***n*****=169 722**	***n*****=150 386**	***n*****=150 386**	***n*****=158 024**	***n*****=149 693**	***n*****=160 256**	***n*****=140 749**
**Incident CRC**	***n*****=761**	***n*****=946**	***n*****=685**	***n*****=961**	***n*****=965**	***n*****=830**	***n*****=830**	***n*****=884**	***n*****=825**	***n*****=898**	***n*****=764**
**Top 10%**											
Sensitivity	13.8	20.2	21.5	11.1	12.2	22.5	24.7	24.9	26.4	14.5	22.6
Specificity	90.0	90.1	90.1	90.0	90.0	90.1	90.1	90.1	90.1	90.0	90.1
LR+	1.38	2.03	2.16	1.11	1.22	2.27	2.49	2.51	2.67	1.45	2.28
LR−	0.96	0.89	0.87	0.99	0.98	0.86	0.84	0.83	0.82	0.95	0.86
PPV (%)	0.8	1.1	1.4	0.6	0.7	1.2	1.4	1.4	1.5	0.8	1.2
NPV (%)	99.5	99.5	99.4	99.4	99.4	99.5	99.5	99.5	99.5	99.5	99.5
**Top 20%**											
Sensitivity	25.8	38.5	35.3	29.9	23.2	40.0	42.8	42.8	41.3	23.3	36.5
Specificity	80.0	80.1	80.1	80.1	80.0	80.1	80.1	80.1	80.1	80.0	80.1
LR+	1.29	1.93	1.78	1.50	1.16	2.01	2.15	2.15	2.08	1.16	1.83
LR−	0.93	0.77	0.81	0.88	0.96	0.75	0.71	0.71	0.73	0.96	0.79
PPV (%)	0.7	1.1	1.2	0.8	0.7	1.1	1.2	1.2	1.1	0.7	1.0
NPV (%)	99.5	99.6	99.5	99.5	99.5	99.6	99.6	99.6	99.6	99.5	99.6
**Top 80%**											
Sensitivity	86.2	95.2	90.7	96.1	71.4	97.0	96.7	97.1	95.6	84.2	85.9
Specificity	20.0	20.1	20.1	20.1	20.0	20.1	20.1	20.1	20.1	20.0	20.0
LR+	1.08	1.19	1.13	1.20	0.89	1.21	1.21	1.21	1.20	1.05	1.07
LR−	0.69	0.24	0.47	0.19	1.43	0.16	0.16	0.15	0.22	0.79	0.71
PPV (%)	0.6	0.7	0.8	0.7	0.5	0.7	0.7	0.7	0.7	0.6	0.6
NPV (%)	99.6	99.9	99.7	99.9	99.2	99.9	99.9	99.9	99.9	99.6	99.6
**Top 90%**											
Sensitivity	94.3	98.0	96.6	99.1	82.7	98.8	99.0	99.1	97.5	91.4	88.7
Specificity	10.0	10.0	10.0	10.1	10.0	10.0	10.1	10.1	10.0	10.0	10.0
LR+	1.05	1.09	1.07	1.10	0.92	1.10	1.10	1.10	1.08	1.02	0.99
LR−	0.56	0.20	0.33	0.09	1.74	0.12	0.10	0.09	0.25	0.86	1.13
PPV (%)	0.6	0.6	0.7	0.6	0.5	0.6	0.6	0.6	0.6	0.6	0.5
NPV (%)	99.7	99.9	99.8	99.9	99.0	99.9	99.9	99.9	99.8	99.5	99.4

Abbreviations: LR+=positive likelihood ratio; LR−=negative likelihood ratio; NPV=negative predictive value; PPV=positive predictive value.

**Table 4:** Discriminatory performance measures for each of the risk models for 5 year risk of developing colorectal cancer in women

	**Colditz**	**Freedman**	**Guesmi**	**Johnson**	**QCancer10**	**Tao**	**Wei Y-S**	**Wells**	**Driver**^a^	**Ma (simple)**^a^	**Ma (cox)**^a^
Total n	*n*=164 034	*n*=130 188	*n*=202 620	*n*=203 390	*n*=193 365	*n*=189 097	*n*=194 601	*n*=191 475	*n*=201 474	*n*=174 297	*n*=174 297
Incident CRC	*n* = 592	*n* = 562	*n* = 752	*n* = 754	*n* = 714	*n* = 696	*n* = 716	*n* = 713	*n* = 745	*n* = 628	*n* = 628
**Top 10%**											
Sensitivity	12.3	16.5	11.2	19.8	19.0	18.0	11.0	18.9	17.4	19.6	19.3
Specificity	90.0	90.0	90.0	90.0	90.0	90.0	90.0	90.0	90.0	90.0	90.0
LR+	1.23	1.66	1.12	1.00	1.91	1.80	1.10	1.90	1.75	1.97	1.93
LR−	0.97	0.93	0.99	0.98	0.90	0.91	0.99	0.90	0.92	0.89	0.90
PPV (%)	0.4	0.7	0.4	0.4	0.7	0.7	0.4	0.7	0.6	0.7	0.7
NPV (%)	99.6	99.6	99.6	99.6	99.7	99.7	99.6	99.7	99.7	99.7	99.7
**Top 20%**											
Sensitivity	21.5	30.1	28.3	22.9	36.1	33.9	19.3	33.1	30.9	31.2	32.6
Specificity	80.0	80.0	80.0	80.0	80.1	80.1	80.0	80.0	80.0	80.0	80.0
LR+	1.07	1.51	1.42	1.15	1.81	1.70	1.01	1.66	1.55	1.56	1.64
LR−	0.98	0.87	0.90	0.96	0.80	0.83	0.95	0.84	0.86	0.86	0.84
PPV (%)	0.4	0.6	0.5	0.4	0.7	0.6	0.4	0.6	0.6	0.6	0.6
NPV (%)	99.6	99.6	99.7	99.6	99.7	99.7	99.6	99.7	99.7	99.7	99.7
**Top 80%**											
Sensitivity	80.2	86.1	92.7	76.5	93.6	91.4	78.2	93.1	91.9	93.0	92.0
Specificity	20.0	20.0	20.0	20.0	20.1	20.0	20.0	20.0	20.0	20.0	20.0
LR+	1.00	1.08	1.16	0.96	1.17	1.14	0.98	1.16	1.15	1.16	1.15
LR−	0.99	0.69	0.36	1.17	0.32	0.43	1.09	0.34	0.40	0.35	0.40
PPV (%)	0.4	0.5	0.4	0.4	0.4	0.4	0.4	0.4	0.4	0.4	0.4
NPV (%)	99.6	99.7	99.9	99.6	99.9	99.8	99.6	99.9	99.9	99.9	99.9
**Top 90%**											
Sensitivity	90.4	94.0	97.2	88.2	97.1	95.1	88.8	96.9	95.7	96.7	95.7
Specificity	10.0	10.0	10.0	10.0	10.0	10.0	10.0	10.0	10.0	10.0	10.0
LR+	1.00	1.04	1.08	0.98	1.08	1.06	0.99	1.08	1.06	1.07	1.06
LR−	0.96	0.60	0.27	1.18	0.29	0.49	1.12	0.31	0.43	0.33	0.43
PPV (%)	0.4	0.5	0.4	0.4	0.4	0.4	0.4	0.4	0.4	0.4	0.4
NPV (%)	99.6	99.7	99.9	99.6	99.9	99.8	99.6	99.9	99.8	99.9	99.8

Abbreviations: LR+=positive likelihood ratio; LR−=negative likelihood ratio; NPV=negative predictive value; PPV=positive predictive value.

a Models originally developed only for men.
