# Correction: External validation of risk prediction models for incident colorectal cancer using UK Biobank

**DOI:** 10.1038/s41416-020-0767-0

**Published:** 2020-03-12

**Authors:** J. A. Usher-Smith, A. Harshfield, C. L. Saunders, S. J. Sharp, J. Emery, F. M. Walter, K. Muir, S. J. Griffin

**Affiliations:** 1 The Primary Care Unit, Department of Public Health and Primary Care, University of Cambridge, Cambridge, CB2 0SR UK; 2 MRC Epidemiology Unit, University of Cambridge, Institute of Metabolic Science, Cambridge, CB2 0QQ UK; 3 Department of General Practice, Centre for Cancer Research, Faculty of Medicine, Dentistry and Health Sciences, The University of Melbourne, Victorian Comprehensive Cancer Centre, Melbourne, VIC 3010 Australia; 4 Institute of Population Health, University of Manchester, Manchester, M13 9PL UK

**Correction to:** *British Journal of Cancer* (2018) **118**, 750–759; 10.1038/bjc.2017.463, published online 30 January 2018.

Since the publication of this paper, the authors have identified an error in the code they used in Stata to compute the Wells risk score for men. With the correct code, the performance of the Wells risk score is improved. The correct values are included in the updated versions of Table 3, Fig. 1 (Fig. 1A), Fig. 2 (Fig. 2A) and Supplementary Table 3 provided here. The Wells risk score is now one of the best performing models in men as well as in women. This does not change the overall conclusions of the analysis but in all places in the paper where reference is made to the best performing models in men, the correct list is Tao, Drive, Ma and Wells.

**Table 3:** Discriminatory performance measures for each of the risk models for 5 year risk of developing colorectal cancer in men.

	**Colditz**	**Driver**	**Freedman**	**Guesmi**	**Johnson**	**Ma (simple)**	**Ma (Cox)**	**QCancer10**	**Tao**	**Wei Y-S**	**Wells**
Total n	*n*=139,257	*n*=167,762	*n=*101,530	*n*=168,825	*n*=169,722	*n*=150,386	*n*=150,386	*n*=158,024	*n*=149,693	*n*=160,256	*n*=140,917
Incident CRC	*n* =761	*n* = 946	*n* = 685	*n* = 961	*n* = 965	*n* = 830	*n* = 830	*n* = 884	*n* = 825	*n* = 898	*n* = 764
Top 10%											
Sensitivity	13.8	20.2	21.5	11.1	12.2	22.5	24.7	24.9	26.4	14.5	28.7
Specificity	90.0	90.1	90.1	90.0	90.0	90.1	90.1	90.1	90.1	90.0	90.1
LR+	1.38	2.03	2.16	1.11	1.22	2.27	2.49	2.51	2.67	1.45	2.90
LR-	0.96	0.89	0.87	0.99	0.98	0.86	0.84	0.83	0.82	0.95	0.79
PPV (%)	0.8	1.1	1.4	0.6	0.7	1.2	1.4	1.4	1.5	0.8	1.6
NPV (%)	99.5	99.5	99.4	99.4	99.4	99.5	99.5	99.5	99.5	99.5	99.6
Top 20%											
Sensitivity	25.8	38.5	35.3	29.9	23.2	40.0	42.8	42.8	41.3	23.3	45.3
Specificity	80.0	80.1	80.1	80.1	80.0	80.1	80.1	80.1	80.1	80.0	80.1
LR+	1.29	1.93	1.78	1.50	1.16	2.01	2.15	2.15	2.08	1.16	2.28
LR-	0.93	0.77	0.81	0.88	0.96	0.75	0.71	0.71	0.73	0.96	0.68
PPV (%)	0.7	1.1	1.2	0.8	0.7	1.1	1.2	1.2	1.1	0.7	1.2
NPV (%)	99.5	99.6	99.5	99.5	99.5	99.6	99.6	99.6	99.6	99.5	99.6
Top 80%											
Sensitivity	86.2	95.2	90.7	96.1	71.4	97.0	96.7	97.1	95.6	84.2	97.1
Specificity	20.0	20.1	20.1	20.1	20.0	20.1	20.1	20.1	20.1	20.0	20.1
LR+	1.08	1.19	1.13	1.20	0.89	1.21	1.21	1.21	1.20	1.05	1.22
LR-	0.69	0.24	0.47	0.19	1.43	0.16	0.16	0.15	0.22	0.79	0.14
PPV (%)	0.6	0.7	0.8	0.7	0.5	0.7	0.7	0.7	0.7	0.6	0.7
NPV (%)	99.6	99.9	99.7	99.9	99.2	99.9	99.9	99.9	99.9	99.6	99.9
Top 90%											
Sensitivity	94.3	98.0	96.6	99.1	82.7	98.8	99.0	99.1	97.5	91.4	99.1
Specificity	10.0	10.0	10.0	10.1	10.0	10.0	10.1	10.1	10.0	10.0	10.0
LR+	1.05	1.09	1.07	1.10	0.92	1.10	1.10	1.10	1.08	1.02	1.10
LR-	0.56	0.20	0.33	0.09	1.74	0.12	0.10	0.09	0.25	0.86	0.09
PPV (%)	0.6	0.6	0.7	0.6	0.5	0.6	0.6	0.6	0.6	0.6	0.6
NPV (%)	99.7	99.9	99.8	99.9	99.0	99.9	99.9	99.9	99.8	99.5	100

*LR+* positive likelihood ratio; *LR−* negative likelihood ratio; *PPV* positive predictive value; *NPV* negative predictive value.

**Fig. 1:**
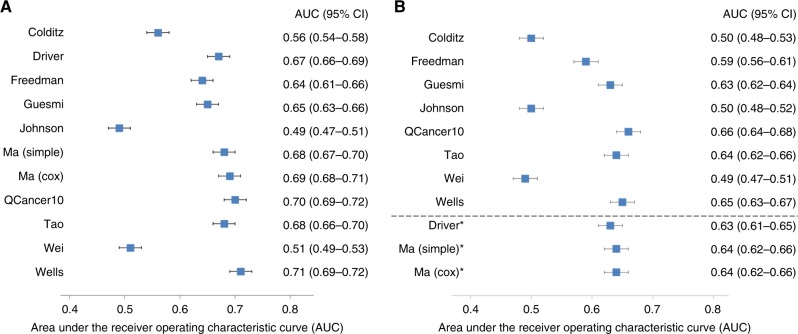
Model discrimination. Area under the receiver operating characteristic curve for the risk models in (**A**) men and (**B**) women. *Models originally only developed in men.

**Fig. 2:**
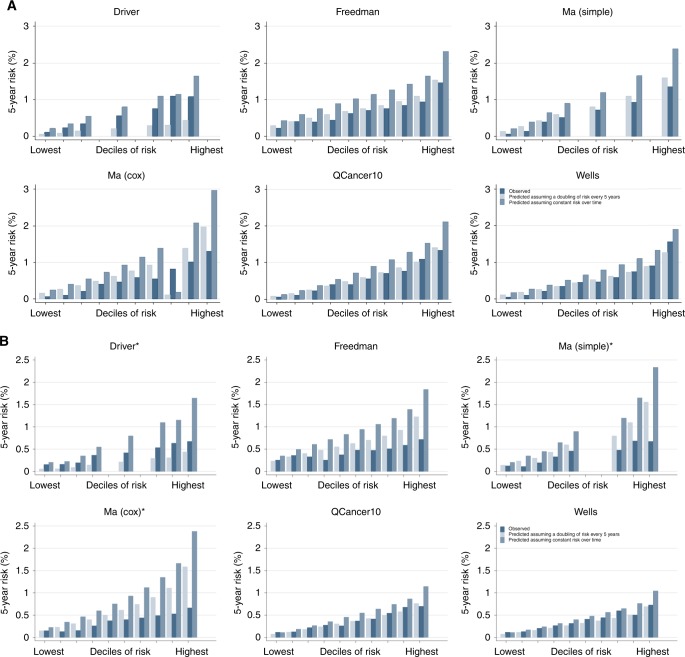
Model calibration. Plots of observed and predicted 5-year risk of colorectal cancer for (**A**) men and (**B**) women. *Models originally only developed in men.

## Supplementary information

Supplemental Material

