# Supplementary figures and images for: External validation of risk prediction models for incident colorectal cancer using UK Biobank
Source: Br J Cancer. 2018 Jan 30;118(5):750–9. doi: 10.1038/bjc.2017.463 (PMC5846069; doi:10.1038/bjc.2017.463)

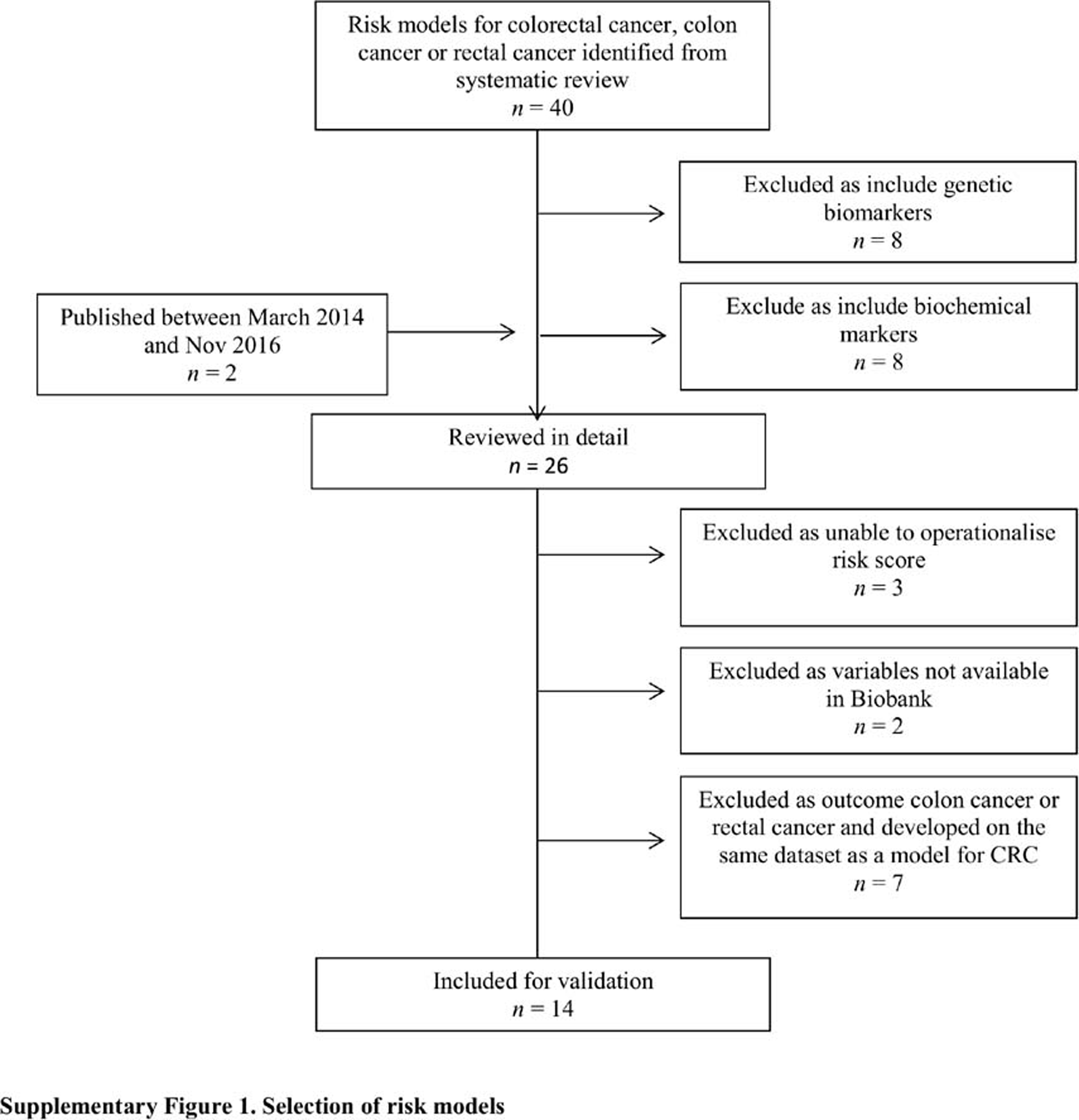

Supplement: Supplementary Figure 1 [file bjc2017463x1.tif]

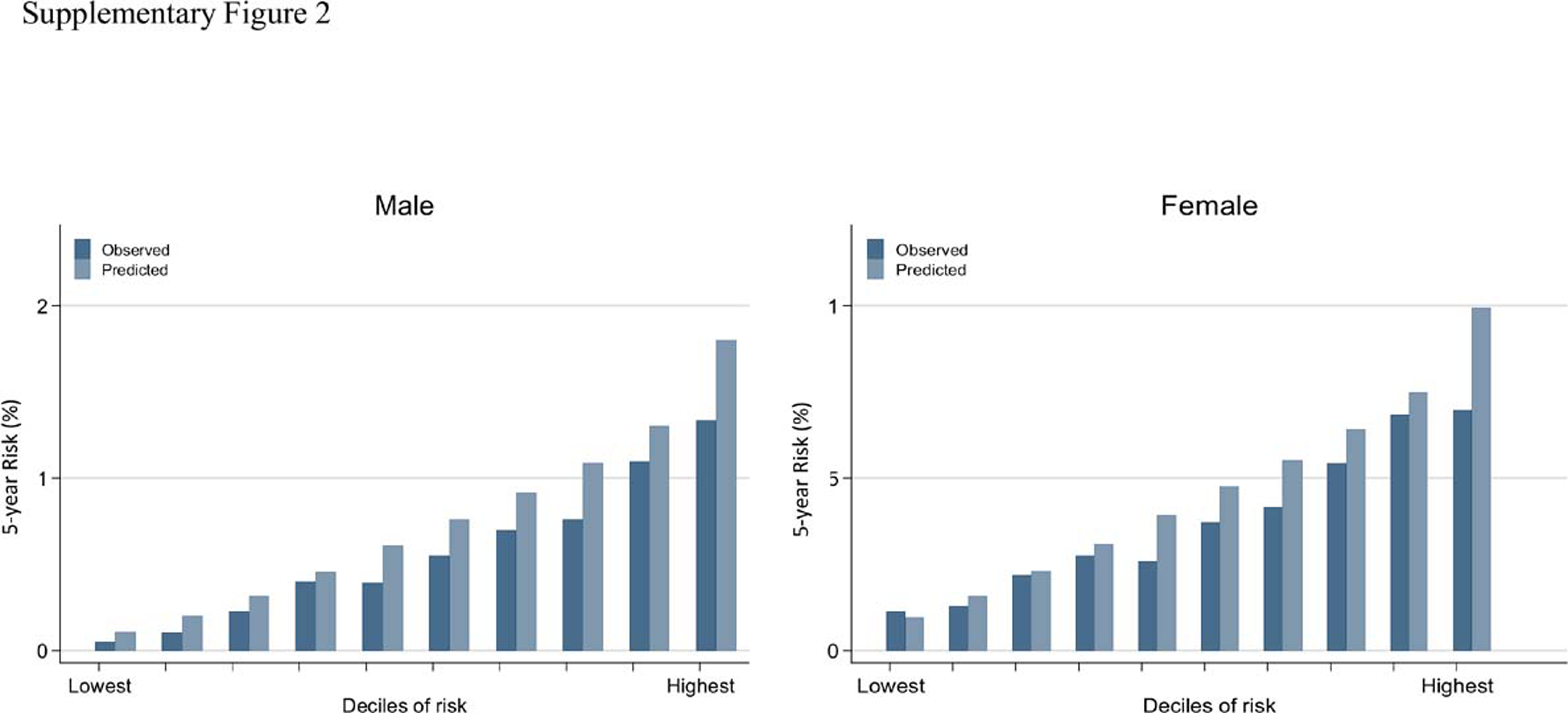

Supplement: Supplementary Figure 2 [file bjc2017463x2.tif]
