# Supplementary material for: External validation of risk prediction models for incident colorectal cancer using UK Biobank
Source: Br J Cancer. 2018 Jan 30;118(5):750–9. doi: 10.1038/bjc.2017.463 (PMC5846069; doi:10.1038/bjc.2017.463)
Supplement: Supplementary Table 1 [file bjc2017463x3.pdf]

Supplementary Table 1 – Full equations for incident colorectal cancer risk models

**Colon cancer**
Colditz 2000 simple point score model
The risk score was calculated as X; Where<br>X = 10 (if family history of colorectal cancer, else 0) + 10 (if BMI ≥ 27/km/m^2, else 0) + 5 (if alcohol intake ≥8 servings per week, else 0) + 5 (if vegetable portions < 3 per week, else 0) + 5 (if height ≥ 5 ft 7 in, else 0) + 10 (if total leisure physical activity time < 3 hours per week, else 0) + 10 (if red meat consumption ≥ 3 per week, else 0) + 5 (if use of birth control pills < 5 years, else 0) + 5 (if use of postmenopausal hormones < 5 years, else 0) + 5 (if currently use aspirin on a regular basis, else 0) + 10 (if not regularly using multivitamins, else 0) + 10 (if has history of inflammatory bowel disease for ≥ 10 years, else 0) + 5 (if consume milk or dairy products (saturated fats) ≥3 servings per day, else 0) + 10 (if do not take calcium supplement every day, else 0) + 10 (if do not take vitamin D supplement every day, else 0)
**Colorectal cancer**
Driver 2007 simple point score model
The risk score was calculated as X; Where<br>X = age score + BMI score + smoking status score + alcohol intake score<br>Where<br>Age score = 2 if 50-59 years; 4 if 60-69 years; 6 if ≥70<br>BMI score = 1 if 25.0-29.9; 2 if ≥30<br>Smoking status score = 1 if smoker; 0 if else<br>Alcohol intake score = 1 if ≥once/week; 0 if else
Freedman 2009 logistic model
The probability of developing colorectal cancer was calculated using the Gauss Program available at https://dceg.cancer.gov/tools/risk-assessment/ccratgauss
Guesmi 2010 logistic model
The risk score was calculated as X; Where<br>X = (0.082 * age) + 1.631 (if consume processed meat frequently, else 0) - 1.952 (if consume milk frequently, else 0)
Johnson 2013 restricted cubic spine model
Assuming multiplicative joint effects between all risk factors included in the meta-analysis, selecting the “Overall” estimate where multiple estimates are provided, the risk score was calculated as the baseline risk among people with the lowest risk levels of all risk factors multiplied by X; where exp denotes the exponential function and where<br>X = exp (0.032 x (BMI-22)/8, if male, else 0) x exp(0.017 x (BMI-22)/8, if female, else 0) x exp(-0.061 x standardised physical activity) x exp(0.011 x pack-years - 0.017 x rcs) x exp(0.011x drinks/week) x exp(-0.120 x years of current HT use + 0.415 x rcs, if current HRT user, else 0) x exp(-0.008 x years of former HRT use, if former HRT user) x exp(-0.069 x years of aspirin use + 0.146 x rcs) x exp(0.017 x servings of processed meat per week) x exp(0.024 x servings of red meat per week) x exp(-0.104 x servings/day + 0.128 x rcs) x 1.80, if CRC family history, else 1 x 2.93 if IBD, else 1<br>Where: $rcs = (dose - knot_1)_+^3 - \frac{\frac{(knot_3 - knot_1)(dose - knot_2)_+^3 - (knot_2 - knot_1)(dose - knot_3)_+^3}{knot_3 - knot_2}}{(knot_3 - knot_1)^2}$<br>For smoking knots = (0, 14.2, 57.7), for current HRT use knots = (0, 1.7, 14.4), for fruit consumption knots = (0, 1.08, 3.95), for years of aspirin use (0, 2.0, 13.2) and “dose” = the risk factor unit in the model (pack years for smoking, years of HRT use and serving of fruit per day)
Ma 2010 simple point score model
The risk score was calculated as X; Where<br>X = age score + BMI score + smoking status score + alcohol intake score + physical activity score<br>Where<br>Age score = 0 if 40-44 years; 1 if 45-49 years; 3 if 50-54 years; 4 if 50-59 years; 5 if 60-64 years; 6 if 65-69 years<br>BMI score = 0 if <25kg/m^2; 1 if ≥25kg/m^2

Smoking status score = 0 if non-smoker or former smoker; 1 if current smoker
Alcohol intake score = 0 if never or occasional; 1 if regular <300g/week; 2 if regular ≥300g/week
Physical activity score = 0 if <24.7 MET-h/day; -1 if ≥24.7 MET-h/day

Ma 2010 cox proportional hazards model

The probability of developing colorectal cancer was calculated as 1- $S(t)^{\exp(f[x,M])}$; Where
$S(t)$ = 0.9882
f(x,M) = ((0.080 * (individual's age – 52.9)) + ((0.04 * (individual's BMI – 23.4)) + ((-0.019 * (individual's physical activity – 28.7)) + (0 * (1 (if never smoker, else 0)) + (0.071 * (1 (if ex-smoker, else 0) – 0.239)) + (0.239 * (1 (if current smoker, else 0) – 0.525)) + (-0.163 * (1 (if never drinks alcohol, else 0) – 0.235)) + (0 * (1 (if occasionally drinks alcohol, else 0)) + (0.358 * 1 (if regular alcohol consumption <300g/w, else 0) – 0.481)) + (0.659 * (1 (if regular alcohol consumption ≥300g/w, else 0) – 0.208))

Tao 2014 simple point score model

The risk score was calculated as X; Where
X = (6 * individual's age) + (104 * (1 (if male, else 0)) + (35 * number of first degree relatives with bowel cancer) + (1 * pack years of smoking) + (1 * alcohol use g/day) + (-31 * (1 (if ever regular use of NSAID, else 0)) + (-147 * (1 (if previous colonoscopy, else 0)) + (187 * (1 (if history of colonic polyp, else 0)) + (47 * (1 (if red meat consumption > 1 time/day, else 0))

Wei 2009 logistic model

The risk score was calculated as X; Where
X = -0.24846 (if a former smoker) + 0.00995 (if a current smoker) + 0.832909 (if former alcohol intake) + 2.15924 (if current alcohol intake) + 0.482426 (if has family history of colorectal cancer) + 0.329304 (if BMI > 24)

Wells 2015 cox proportional hazards model (male)

The probability of developing colorectal cancer was calculated as 1- $S(t)^{\exp(f[x,M])}$; Where:
$S(t)$=0.9846654
X = (- 6.6419738 (

+ 0.091669179	* (age in years)
- 3.7611814e-05	* (the maximum of age - 47 or 0) ^ 3)
+ 7.794128e-05	* (the maximum of age - 60 or 0) ^ 3)
- 4.0529466e-05	* (the maximum of age - 72 or 0) ^ 3)
- 0.16728044	* (1 if ethnicity is white, else 0)
+ 0.00022581331	* (pack years of smoking)
- 1.1341047e-05	* (the maximum of pack years of smoking or 0) ^ 3)
- 1.3522018e-05	* (the maximum of pack years of smoking – 6.375 or 0) ^ 3)
+ 2.1809706e-06	* (the maximum of pack years of smoking – 39.525 or 0) ^ 3)
+ 0.28379769	* (alcohol intake in drinks per day)
- 0.21424251	* (the maximum of alcohol intake or 0) ^ 3)
+ 0.22570057	* (the maximum of alcohol intake - 0.14457189 or 0) ^ 3)
- 0.011458065	* (the maximum of alcohol intake - 2.8477722 or 0) ^ 3)
+ 0.018020786	* (BMI in kg/m^2)
+ 9.4715899e-05	* (the maximum of BMI - 22.047175 or 0) ^ 3)
- 0.00015791645	* (the maximum of alcohol intake - 25.941735 or 0) ^ 3)
+ 6.3200548e-05	* (the maximum of alcohol intake - 31.778341 or 0) ^ 3)
+ 0.072052428	* (number of years of education)
- 0.00060634342	* (the maximum of the number of years of education – 7 or 0) ^ 3)
+ 0.0016674444	* the maximum of the number of years of education – 14 or 0) ^ 3)
- 0.001061101	* (the maximum of the number of years of education – 18 or 0) ^ 3)
- 0.20960315	* (current aspirin use=1, else 0)
+ 0.24250922	* (family history of colorectal cancer=1, else 0)
- 0.19175375	* (multivitamin use=1, else 0)
+ 0.073141733	* (red meat intake per day)
- 0.0043503755	* (the maximum of the amount of red meat intake - 0.59081962 or 0) ^ 3)
+ 0.0065250851	* (the maximum of the amount of red meat intake - 2.0822052 or 0) ^ 3)

- 0.0021747085 * (the maximum of the amount of red meat intake - 5.0656345 or 0) ^ 3)
+ 0.11020556 * (diabetes=1, else 0)
- 0.090669913 * (moderate or strenuous physical activity in hours per day)
+ 0.0093816671 * (the maximum of the amount of PA activity - 0.10714286 or 0) ^ 3)
- 0.011850527 * (the maximum of the amount of PA activity - 0.82142857 or 0) ^ 3)
+ 0.0024688598 * (the maximum of the amount of PA activity - 3.5357143 or 0) ^ 3)))

Wells 2015 cox proportional hazards model (female)

The probability of developing colorectal cancer was calculated as 1- $S(t)^{exp(f[x,M)}$; Where:
$S(t)$=0.9901043;
X = (- 5.9026635 (
+ (0.090012542 * age in years)
- 4.4217156e-05 * (the maximum of age – 47 or 0) ^ 3)
+ 9.2119076e-05 * (the maximum of age – 60 or 0) ^ 3)
- 4.7901919e-05 * (the maximum of age – 72 or 0) ^ 3)
- 0.34056094 * (1 if ethnicity is white, else 0)
+ 0.07443905 * (number of years of education)
- 0.00062546554 * (the maximum of the number of years of education – 7 or 0) ^ 3)
+ 0.0017200302 * (the maximum of the number of years of education – 14 or 0) ^ 3)
- 0.0010945647 * (the maximum of the number of years of education – 18 or 0) ^ 3)
- 0.2450076 * (current oestrogen use=1, else 0)
- 0.044320489 * (past oestrogen use=1, else 0)
+ 0.23328937 * (diabetes=1, else 0)
+ 0.062703176 * (pack years of smoking)
- 0.002446026 * (the maximum of pack years of smoking or 0)^3)
+ 0.003038396 * (the maximum of pack years of smoking - 1.25 or 0) ^ 3)
- 0.00059134632 * (the maximum of pack years of smoking - 6.375 or 0) ^ 3)
- 1.023614e-06 * (the maximum of pack years of smoking - 27.5125 or 0) ^ 3)
+ 0.31589053 * (family history of colorectal cancer=1, else 0)
- 0.1665365 * (multivitamin use=1, else 0)
+ 0.0075233925 * (BMI in kg/m^2)
+ 6.7918662e-05 * (the maximum of BMI - 20.371336 or 0) ^ 3)
- 0.00011039091 * (the maximum of BMI - 25.508027 or 0) ^ 3)
+ 4.2472244e-05 * (the maximum of BMI - 33.722266 or 0) ^ 3)
- 0.236997 * (current NSAID use=1, else 0)
- 0.08856241 * (alcohol intake in drinks per day)
+ 0.62375456 * (the maximum of alcohol intake or 0) ^ 3)
- 0.7191129 * (the maximum of alcohol intake - 0.10740682 or 0) ^ 3)
+ 0.095358349 * (the maximum of alcohol intake - 0.8099724 or 0) ^ 3)))

Hippisley-Cox 2015 cox proportional hazards model (QCancer10)* (male)

The probability of developing colorectal cancer within 10 years among men was calculated as 1 – $S_0(10)^{exp(X)}$ where exp denotes the exponential function and where:
$S_0(10)$= 0.997250497341156
and
X= $X_{categorical} + X_{continuous} + X_{Boolean} + X_{Interaction}$
$X_{categorical} = X_{alcohol} + X_{ethnicity} + X_{smoking}$
$X_{alcohol}$ = 0 if alcohol intake = none, else
$X_{alcohol}$ = 0.047922717327152427 if alcohol intake = <1 unit / day, else
$X_{alcohol}$ = 0.1306450076527492 if alcohol intake = 1-2 units/day, else
$X_{alcohol}$ = 0.26424169760665095 if alcohol intake = 3-6 units / day, else
$X_{alcohol}$ = 0.48248078623669538 if alcohol intake = 7-9 units / day, else
$X_{alcohol}$ = 0.44338870524869628 if alcohol intake = >9 units / day
$X_{ethnicity}$ = 0 if ethnicity = White or not stated
$X_{ethnicity}$ =-0.58523471759902834 if ethnicity = Indian
$X_{ethnicity}$ =-0.58643377805068653 if ethnicity = Pakistani

$X_{ethnicity}$ =-0.88110809361453912 if ethnicity = Bangladeshi
$X_{ethnicity}$ =-0.4825211311076027 if ethnicity = Other Asian
$X_{ethnicity}$ =-0.35029857021954686 if ethnicity = Black caribbean
$X_{ethnicity}$ =-0.28686430423891179 if ethnicity = Black African
$X_{ethnicity}$ =-0.21258459900822296 if ethnicity = Chinese
$X_{ethnicity}$ =-0.5250625721689498 if ethnicity = Other
$X_{smoking}$ = 0 if smoking status= never smoker
$X_{smoking}$ = 0.054436520378164946 if smoking status=ex-smoker
$X_{smoking}$ = 0.066967983943209852 if smoking status=light smoker (1-9 per day)
$X_{smoking}$ = 0.026825493057743323 if smoking status=moderate smoker (10-19 per day)
$X_{smoking}$ = 0.12301347198058288 if smoking status=heavy smoker (20+ per day)
$X_{continuous}$ = age_1 x 2.6296558591088894 + age_2 x -0.14957281392190663 + BMI x 0.015645211942720651 + Town x 0.0089992920978670451
$X_{Boolean}$= H/O blood cancer (1 if present, else 0) x 0.42840787527086416 + H/O colitis (1 if present, else 0) x 0.60167262208697581 + H/O Lung Cancer (1 if present, else 0) x 0.62731856184500678 + H/O Oral cancer (1 if present, else 0) x 0.48251770458128385 + H / O Colorectal polyps (1 if present, else 0) x 0.4092716863938517 + H / O Type 2 diabetes (1 if present, else 0) x 0.23892082443419493 + Family History of bowel cancer (1 if present, else 0) x 0.78029895261674453
$X_{interaction}$ = age_1 x Family History of bowel cancer (1 if present, else 0) x -1.6277021020802243 + age_2 x Family History of bowel cancer (1 if present, else 0) x 0.12483775307131491
and
age_1= age/10 - 4.425716876983643
age_2=$(age/10)^2$ - 19.586969375610352
BMI=BMI- 26.309040069580078
Town=Townsend Score - 0.260672301054001

Hippisley-Cox 2015 cox proportional hazards model (QCancer10)* (female)

The probability of developing colorectal cancer within 10 years among women was calculated as 1 – $S_0(10)^{exp(X)}$ where exp denotes the exponential function and where:
$S_0(10)$= 0.997171759605408
and
X= $X_{categorical}$ + $X_{continuous}$ + $X_{Boolean}$ + $X_{Interaction}$
$X_{categorical}$ = $X_{alcohol}$ + $X_{ethnicity}$ + $X_{smoking}$
$X_{alcohol}$ = 0 if alcohol intake = none, else
$X_{alcohol}$ = 0.016932317022484617 if alcohol intake = <1 unit / day, else
$X_{alcohol}$ = 0.050471611879332329 if alcohol intake = 1-2 units/day, else
$X_{alcohol}$ = 0.081302218350274985 if alcohol intake = 3-6 units / day, else
$X_{alcohol}$ = 0.31884276410032175 if alcohol intake = 7-9 units / day, else
$X_{alcohol}$ = 0.30711384933305064 if alcohol intake = >9 units / day
$X_{ethnicity}$ = 0 if ethnicity = White or not stated
$X_{ethnicity}$ =--1.0494971746256023 if ethnicity = Indian
$X_{ethnicity}$ =-0.75800858721677611 if ethnicity = Pakistani
$X_{ethnicity}$ =-0.16206418242822976 if ethnicity = Bangladeshi
$X_{ethnicity}$ =-0.52075515892893942 if ethnicity = Other Asian
$X_{ethnicity}$ =-0.3390081073116003 if ethnicity = Black caribbean
$X_{ethnicity}$ =-0.36639919625711181 if ethnicity = Black African
$X_{ethnicity}$ =-0.49625527321514168 if ethnicity = Chinese
$X_{ethnicity}$ =-0.22289770148509797 if ethnicity = Other
$X_{smoking}$ = 0 if smoking status= never smoker
$X_{smoking}$ = 0.067943065824236953 if smoking status=ex-smoker
$X_{smoking}$ = 0.10017056701706135 if smoking status=light smoker (1-9 per day)
$X_{smoking}$ = 0.19403251493802176 if smoking status=moderate smoker (10-19 per day)
$X_{smoking}$ = 0.16095242485295916 if smoking status=heavy smoker (20+ per day)
$X_{continuous}$ = age_1 x 33.92788234800954 + age_2 x --83.924050851993556
$X_{Boolean}$= H/O breast cancer (1 if present, else 0) x 0.15096136873617999 + H/O cervical cancer (1 if present, else 0) x 0.55134659369582772 + H/O colitis (1 if present, else 0) x 0.56087545120009641 +

H/O ovarian cancer (1 if present, else 0) x 0.68400222067217209 + H / O Colorectal polyps (1 if present, else 0) x 0.74904091187947031 + H / O Type 2 diabetes (1 if present, else 0) x 0.14828780874778763 + H/O endometrial cancer (1 if present, else 0) x 0.47923953865647911 + Family History of bowel cancer (1 if present, else 0) x 0.66160452485446319

$X_{interaction}$ = age_1 x Family History of bowel cancer (1 if present, else 0) x 10.985496891972508 + age_2 x Family History of bowel cancer (1 if present, else 0) x -0.094897933584199498

and

age_1= $(age/10)^{-2}$ - 0.049712907522917

age_2=$(age/10)^{-2}$ x log(age/10) - 0.074606411159039

* The beta coefficients not included in the original paper were downloaded in September 2016 from the website of the model using a link obtained from the investigators: http://svn.clinrisk.co.uk/opensource/qcancer/10yr/
