# Supplementary material for: External validation of risk prediction models for incident colorectal cancer using UK Biobank
Source: Br J Cancer. 2018 Jan 30;118(5):750–9. doi: 10.1038/bjc.2017.463 (PMC5846069; doi:10.1038/bjc.2017.463)
Supplement: Supplementary Table 2 [file bjc2017463x4.pdf]

Supplementary Table 2 - Derivation of risk scores variables

**Age:** For all models including age either as a categorical or continuous variable the age of participants when they first attended the Biobank assessment centre was used.
**Sex:** For all models including sex, sex at baseline was used to categorise participants into male and female.
**Ethnicity:** The Wells models for both men and women include a predictor with ethnicity defined as Hawaiian, Japanese, Latino or White and the Freedman models included categories for White, African-American, Hispanic and Asian-American Due to the predominantly white population in UK Biobank and the fact that the ethnicity data collected in the Biobank cohort did not include separate categories for many of these groups, these variables were collapsed into two categories: white or other for both models.
**BMI:** The Biobank variable for BMI as constructed from height and weight measured during the initial assessment centre visit was used in all models including BMI as either a categorical or continuous variable.
**Family history:** Most of the models which included family history did so with a categorical variable (yes/no). Tao(Tao *et al*, 2014) included a variable for the number of first degree relatives with bowel cancer. Biobank collected data only on whether the participants' mother, father or any of their siblings have been affected by bowel cancer. A positive response for any was considered as a positive family history and any siblings having been affected was treated as one first degree relative.
**Smoking:** Pack years of smoking were included in the Johnson(Johnson *et al*, 2013), Tao(Tao *et al*, 2014) and Wells(Wells *et al*) models. These were calculated from the age started smoking and (if relevant for ex-smokers) the age stopped smoking variables and the number of cigarettes smoked per day (in packs of 20). Pack years are estimated as the number of packs of cigarettes smoked per day multiplied by the number of years of smoking. A reduction of 6 months from the length of time smoking for people who reported that they had previously quite smoking, but had then returned to smoking, and people who reported smoking "less than 1" cigarette a day were coded as 0.5 cigarettes per day for this calculation. People who reported cigar or pipe smoking had pack years coded as missing; people who reported having smoked only "occasionally" or "once or twice" were coded with 0 pack years. The same approach was used for the Freedman model(Freedman *et al*, 2009) which included separate variables for years of smoking and number of cigarettes smoked. For other models (Driver(Driver *et al*, 2007), Ma(Ma *et al*, 2010), Wei(Wei *et al*, 2009), QCancer(Hippisley-Cox & Coupland, 2015)) smoking was incorporated as a binary or categorical variable using the baseline smoking status variable in Biobank (current, ex-smoker, never smoker).
**Alcohol:** Alcohol consumption was defined either as a categorical variable (rarely or never/once or more per week in the Driver model(Driver *et al*, 2007) and never/occasional/regular <300g per week/≥300g per week in the Ma model(Ma *et al*, 2010)), or as a continuous variable as grams/day (Tao model(Tao *et al*, 2014)), units/day (Wells model(Wells *et al*)), or drinks/week (Johnson(Johnson *et al*, 2013)). Consumption of alcohol was collected in Biobank using a screening question 'How often do you drink alcohol?' with responses 'Daily or almost daily', 'three or four times a week', 'once or twice a week', 'one to three times a month', 'special occasions only', 'never'. We defined rarely or occasional as 'one to three times a month' or 'special occasional only'. To calculate intake as units or grams per week we used the responses to detailed questions about weekly and monthly consumption of different alcoholic drinks and converted the number of drinks within each category to units(NHS Choices Livewell Alcohol Units) and then multiplied by 8 (the number of grams per unit in the UK)(UK Parliament Alcohol Guidelines). Where participants were asked about their consumption of a range of different alcohols we assigned a value of zero where data was missing.
**Physical activity:** Physical activity in the Ma(Ma *et al*, 2010) and Johnson(Johnson *et al*, 2013) models was defined in terms of MET-hours. For the Ma model physical activity is reported as METs per 24 hours and hours of activity were converted to MET-hours using the multipliers used in the published paper and responses to questions about physical activity duration. Moderate or vigorous activity frequency (in hours) was multiplied by 4.5, time spent walking by 2, sedentary time (consistent with the approach taken in a previous study using UK Biobank(Celis-Morales *et al*, 2016) including time spent in front of the computer, watching TV or driving) by 1.5 and all other time

(including sleep) by 0.9. Consistent with the approach taken in that paper, where individuals had "missing" hours within the day not accounted for by reported physical activity or sedentary activity, any "missing" hours were coded as sleep/other. For the individuals with more than 24 hours of activity we decreased activity and sedentary time in proportion to the total number of hours in the day that they reported data for. In the Johnson model(Johnson *et al*, 2013) a standardised physical activity score is used, and so we used the IPAQ scoring protocol for walking, moderate and vigorous physical activity, and then standardised this score to a mean of 0 and an SD of 1 (as the biobank questions were designed to be used with this score). In both models people who reported <10 minutes of each type of activity were recoded as 10 minutes. In the Colditz(Colditz *et al*, 2000), Freedman(Freedman *et al*, 2009) and Wells(Wells *et al*) models physical activity was included as either a categorical or continuous variable for number of hours of activity. We used responses to questions about frequency and duration of different levels of physical activity to derive those.
**Red meat consumption**: We considered beef, pork and lamb as red meat(World Health Organisation Q&A on the carcinogenicity of the consumption of red meat and processed meat). The Tao model(Tao *et al*, 2014) included a categorical variable (more than once per day (yes/no)), the Colditz model a categorical variable (3 or more servings per week (yes/no)), the Wells model(Wells *et al*) a continuous variable as ounces per day and the Johnson model(Johnson *et al*, 2013) a continuous variable as servings per week. For each of beef, lamb and pork, participants in Biobank had indicated how often they ate them (Never, < once per week, once per week, 2-4 times per week, once or more daily). We used the mid-point for each category to calculate red meat consumption per day as a continuous variable for each participant and multiplied this by 2.5(NHS Choices Livewell Meat) to obtain ounces per day for the Wells model.
**Aspirin and NSAID use:** The Colditz model(Colditz *et al*, 2000) includes a categorical variable for daily use of aspirin or an NSAID for 15 years or more (yes/no), the Tao model(Tao *et al*, 2014) includes a categorical variable for ever regular use of NSAID (yes/no), the Wells model(Wells *et al*) includes three categories (no/yes, not currently/yes, currently), the Johnson model(Johnson *et al*, 2013) uses duration in years of regular use of aspirin or NSAIDs, and the Freedman model(Freedman *et al*, 2009) included categorical (yes/no) variables for regular use of aspirin or NSAIDs and regular use of ibuprofen. As historic data is not available in Biobank, all participants who answered yes for aspirin and/or ibuprofen to the question 'Do you regularly take any of the following? Aspirin, Ibuprofen, Paracetamol, Codeine' or had a code identified by a clinician on the team as indicating aspirin/NSAID/ibuprofen use in the list of current regular treatments were coded as regular or current users. Previous users and non-users were collapsed into one category in the Wells model. As data for duration of use is not available, the mean duration of use was used from the literature(Hoffmeister *et al*, 2007) for all current users.
**Saturated fat:** The Colditz model(Colditz *et al*, 2000) includes a categorical variable for saturated fat consumption (<3/≥3 servings per day. The derived variable in Biobank for saturated fat based on diet by 24-hour recall was only available for less than half of the participants so we instead derived a proxy variable based on responses to questions about frequency of consumption of cheese and type of milk and spread used for all participants. Frequency of cheese is categorised in Biobank into 'never', 'less than once per week', 'once a week', 2-4 times a week', '5-6 times a week', or 'once or more daily' and we used the mid-point for each category to calculate consumption per day as a continuous variable for each participant. For type of milk and spread use, usually consuming full cream milk or butter were treated as one portion of saturated fat each per day and usually consuming semi-skimmed milk or margarine were treated as half a portion of saturated fat per day. Responses were then combined to generate an overall estimate of saturated fat consumption.
**Regular use of multivitamins:** The Wells(Wells *et al*) and Colditz(Colditz *et al*, 2000) models included 'regular use of multivitamins' as a categorical variable (yes/no). Participants were categorised as 'yes' for this if they indicated that they had consumed 'Multivitamin', 'Multivitamin with iron', 'Multivitamin with calcium' or 'Multivitamin with multimineral' yesterday or included any codes for multivitamin use in the list of current regular treatments.
**Vitamin D supplements:** The Colditz model(Colditz *et al*, 2000) included a categorical variable for 'daily vitamin D supplement' (yes/no). Participants were categorised as 'yes' for this if they indicated they had consumed 'Vitamin D' yesterday or included any codes for vitamin D in the list of current

regular treatments.
**Calcium supplements:** The Colditz model(Colditz *et al*, 2000) included a categorical variable for ‘daily calcium supplement’ (yes/no). Participants were categorised as ‘yes’ for this if they indicated they had consumed ‘Multivitamin with calcium’ or ‘Calcium’ yesterday or included any codes for calcium supplements in the list of current regular treatments.
**Education:** The Wells models(Wells *et al*) included a continuous variable for number of years spent in education. The equivalent variable in Biobank was the age at which participants completed their continuous full time education. The number of years spent in education was computed by subtracting 5 (the age children start UK primary education) from the age at which participants completed their full time education. All those who had a value of less than 0 were set to 0.
**Oestrogen use:** The Wells model(Wells *et al*) and Freedman model(Freedman *et al*, 2009) for females included categorical variables for oestrogen use (current user/past user or regular/non-user). We generated proxy variables for these based on the responses from participants in Biobank to six questions. First we used the responses to “Have you ever used HRT?” and “When did you last use HRT?” to identify current and past users for HRT. As participants were only asked when they last used HRT if they had responded ‘yes’ to the question “Have you ever used HRT?”, those who answered “Do not know” or “Prefer not to answer” to that question were treated as past users. The two corresponding questions for oral contraceptive pill use were used in the same way to identify current and past users of oral contraceptive pills. It was not possible to distinguish between oestrogen-containing oral contraceptive pills and progesterone-only pills. The Colditz model(Colditz *et al*, 2000) also included categorical variables for use of HRT and birth control pills (<5 vs 5 or more years) and the Johnson model(Johnson *et al*, 2013) years of use. The duration of use was calculated by subtracting the age participants reported stating HRT or the oral contraceptive pill from the age they reported last using HRT or the oral contraceptive pill.
**Milk consumption:** The Guesmi model(Guesmi *et al*, 2010) included a categorical variable (rare/frequent) with frequent defined as four or more times per week and rare less than four. As the variables which denote milk and dairy consumption in the UK Biobank are limited largely to the participant’s consumption of the product “yesterday” (i.e. the day before the questionnaire or interview), there is a large amount of missing data, and we were unable to use these variables. Instead we used the variable which asks “What type of milk do you mainly use?”. Options included “Full cream”, “semi-skimmed”, “skimmed”, “soya”, “other type of milk”, and “never/rarely have milk”. We categorised those selecting “soya”, “other type of milk” or “never/rarely have milk” as rare and the others as frequent.
**Processed meat consumption:** The Guesmi model(Guesmi *et al*, 2010) categorised participants into “Frequent” and “Rare” consumers with frequent defined as four or more times per week and rare less than four. The corresponding variable in Biobank categorises participants into those who consume processed meat “Never”, “< once a week”, “once a week”, “2-4 times a week”, “5-6 times a week”, “once or more daily”. We categorised “5-6 times a week” and “once or more daily” as frequent and the others as rare. The Johnson model(Johnson *et al*, 2013) includes a continuous variable for servings of processed meat per week. We used the mid-point for each category to calculate a continuous variable for each participant
**Vegetables:** For both the Colditz(Colditz *et al*, 2000), Freedman(Freedman *et al*, 2009) and Johnson models(Johnson *et al*, 2013) one portion of vegetables was defined as 3 heaped tablespoons of cooked or raw vegetables(NHS Choices Livewell Portion sizes).
**Height:** The Colditz model(Colditz *et al*, 2000) included a categorical variable for whether participants were 5 foot 7 inches or taller (yes/no). The variable for standing height measured at the first assessment visit was used from the Biobank data to categorise participants.
**Deprivation:** The QCancer10(male) model(Hippisley-Cox & Coupland, 2015) included a continuous variable for the Townsend deprivation index. The Townsend deprivation index at recruitment was used from the Biobank data.
**Previous colonoscopy:** The Tao model(Tao *et al*, 2014) included a categorical variable for whether participants had had a previous colonoscopy and the Freedman model(Freedman *et al*, 2009) a categorical variable for a sigmoidoscopy or colonoscopy in the past 10 years. Data on previous colonoscopy was collected from the Biobank participants during the verbal interview with a nurse at

the baseline assessment. Participants were categorised as having had a previous colonoscopy if they reported having had either a 'colonoscopy/sigmoidoscopy' or a 'ct colonoscopy'.
**Inflammatory bowel disease:** The Johnson model(Johnson *et al*, 2013) included a categorical variable for inflammatory bowel disease (yes/no), the QCancer10 models(Hippisley-Cox & Coupland, 2015) a categorical variable for ulcerative colitis (yes/no), and the Colditz model(Colditz *et al*, 2000) a categorical variable for inflammatory bowel disease for more than 10 years (yes/no). Data on history of these conditions was derived from participant self-report at the verbal interview with a nurse at the Biobank baseline assessment. Inflammatory bowel disease was considered any of 'Inflammatory bowel disease', 'ulcerative colitis' or 'crohns disease'. The date of onset prior to the date of the baseline assessment was used to identify those with the condition for more than 10 years for the Colditz model.
**Previous polyp:** The Tao(Tao *et al*, 2014) , Freedman(Freedman *et al*, 2009) and QCancer10 models(Hippisley-Cox & Coupland, 2015) included a categorical variable for prior colonic polyps (yes/no). For all three risk models self-report of 'rectal or colon adenoma/polyps' at the verbal interview with a nurse at the baseline assessment was used to classify participants.
**Prior cancer:** The QCancer10 models(Hippisley-Cox & Coupland, 2015) included categorical variables for whether participants had a history of a range of different cancers (yes/no). The same ICD9 and ICD10 codes were used as for the development of those models.
**Diabetes:** The QCancer10 models(Hippisley-Cox & Coupland, 2015) included a categorical variable for whether participants had type 2 diabetes (yes/no) and the Wells models a categorical variable for 'diabetes'. The corresponding variable in the Biobank cohort was self-report diagnosis of either 'Diabetes', 'Type 1 diabetes' 'gestational diabetes' or 'Type 2 diabetes' at the interview with a nurse at the baseline assessment. The majority were coded as 'Diabetes' without distinguishing between the various subtypes. Participants with either 'Type 2 diabetes' or 'Diabetes' were therefore included for the QCancer10 models and all four categories for the Wells models(Wells *et al*).
**Handling of missing data:** There is variation across questions within the Biobank baseline assessment in how missing responses had been recorded. For example, for some questions a missing response is "truly missing" (i.e. we do not know whether the response means that a risk factor is present or not), while for others, such as medical history or current medication, the absence of an entry means that the risk factor is absent (i.e. it is appropriate to code these as zero, rather than missing). For each risk factor we checked the original question wording and response coding to ensure that we took the correct approach. Where the calculation of a risk factor variable for a model required the combination of multiple responses from across multiple Biobank baseline survey questions, consistent with other external validation approaches(Dagan *et al*, 2017), we used a combination of practical choices with the over-arching approach to ensure missing values were coded where the missingness was truly uninformative, while minimising missing data by assigning values to missing data in some of the questions included in the combination of responses where appropriate. For example, in coding the physical activity as MET-hours per day the first relevant survey questions ask "In a typical week how many days do you do 10 minutes of moderate PA/vigorous PA/walking?" however some people who respond yes to this question then have a "missing" response to the question about the duration of activity. In this situation where the "In a typical week" question was non-missing and non-zero but the "Duration" question was zero or missing we assigned these people 10 minutes per day (for the number of days stated) in line with the response to the initial question. Doing this, however, meant that people with missing data had at least 10 minutes of exercise while some respondents who did reply to the "Duration" question reported <10 minutes. For consistency we also changed these individuals to 10 minutes.

References

Celis-Morales CA, Lyall DM, Anderson J, Iliodromiti S, Fan Y, Ntuk UE, Mackay DF, Pell JP, Sattar N, Gill JMR (2016) The association between physical activity and risk of mortality is modulated by grip strength and cardiorespiratory fitness: evidence from 498 135 UK-Biobank participants. *Eur Heart J* **38**: ehw249, doi:10.1093/eurheartj/ehw249.

Colditz GA, Atwood KA, Emmons K, Monson RR, Willett WC, Trichopoulos D, Hunter DJ (2000) Harvard report on cancer prevention volume 4: Harvard Cancer Risk Index. *Cancer Causes Control*

11: 477–488.
Dagan N, Cohen-Stavi C, Leventer-Roberts M, Balicer RD (2017) External validation and comparison of three prediction tools for risk of osteoporotic fractures using data from population based electronic health records: retrospective cohort study. *BMJ* i6755, doi:10.1136/bmj.i6755.
Driver JA, Gaziano JM, Gelber RP, Lee I-M, Buring JE, Kurth T (2007) Development of a risk score for colorectal cancer in men. *Am J Med* **120**: 257–263, doi:10.1016/j.amjmed.2006.05.055.
Freedman AN, Slattery ML, Ballard-Barbash R, Willis G, Cann BJ, Pee D, Gail MH, Pfeiffer RM (2009) Colorectal cancer risk prediction tool for white men and women without known susceptibility. *J Clin Oncol* **27**: 686, doi:10.1200/JCO.2008.17.4797.A.
Guesmi F, Zoghlami A, Sghaiier D, Nouira R, Dziri C (2010) Alimentary factors promoting colorectal cancer risk: A prospective epidemiologic study. [French]Les facteurs alimentaires predisposant au risque de cancers colorectaux: Etude epidemiologique prospective. *Tunisie Medicale* **88**: 184–189.
Hippisley-Cox J, Coupland C (2015) Development and validation of risk prediction algorithms to estimate future risk of common cancers in men and women: prospective cohort study. *BMJ Open* **5**: e007825, doi:10.1136/bmjopen-2015-007825.
Hoffmeister M, Chang-claude J, Brenner H (2007) Individual and joint use of statins and low-dose aspirin and risk of colorectal cancer : A population-based case – control study. *Int J Cancer* **1330**: 1325–1330, doi:10.1002/ijc.22796.
Johnson CM, Wei C, Ensor JE, Smolenski DJ, Amos CI, Levin B, Berry D a (2013) Meta-analyses of colorectal cancer risk factors. *Cancer Causes Control* **24**: 1207–1222, doi:10.1007/s10552-013-0201-5.
Ma E, Sasazuki S, Iwasaki M, Sawada N, Inoue M (2010) 10-Year risk of colorectal cancer: development and validation of a prediction model in middle-aged Japanese men. *Cancer Epidemiol* **34**: 534–541, doi:10.1016/j.canep.2010.04.021.
NHS Choices Livewell Alcohol Units http://www.nhs.uk/Livewell/alcohol/Pages/alcohol-units.aspx (accessed: 12/12/2016).
NHS Choices Livewell Meat http://www.nhs.uk/Livewell/Goodfood/Pages/meat.aspx (accessed: 12/12/2016).
NHS Choices Livewell Portion sizes http://www.nhs.uk/Livewell/5ADAY/Pages/Portionsizes.aspx (accessed: 12/12/2016).
Tao S, Hoffmeister M, Brenner H (2014) Development and Validation of a Scoring System to Identify Individuals at High Risk for Advanced Colorectal neoplasms Who Should Undergo Colonoscopy Screening. *Clin Gastroenterol Hepatol* **12**: 478–485.
UK Parliament Alcohol Guidelines https://www.publications.parliament.uk/pa/cm201012/cmselect/cmsctech/1536/153605.htm (accessed: 12/12/2016).
Wei Y-S, Lu J-C, Wang L, Lan P, Zhao H-J, Pan Z-Z, Huang J, Wang J-P (2009) Risk factors for sporadic colorectal cancer in southern Chinese. *World J Gastroenterol* **15**: 2526–2530, doi:10.3748/wjg.15.2526.
Wells BJ, Kattan MW, Cooper GS, Jackson L, Koroukian S ColoRectal Cancer Predicted Risk Online (CRC-PRO) Calculator Using Data from the Multi-Ethnic Cohort Study. *J Am Board Fam Med* **27**: 42–55, doi:10.3122/jabfm.2014.01.130040.
World Health Organisation Q&A on the carcinogenicity of the consumption of red meat and processed meat http://www.who.int/features/qa/cancer-red-meat/en/ (accessed: 12/12/2016).
