# Supplementary material for: External validation of risk prediction models for incident colorectal cancer using UK Biobank
Source: Br J Cancer. 2018 Jan 30;118(5):750–9. doi: 10.1038/bjc.2017.463 (PMC5846069; doi:10.1038/bjc.2017.463)
Supplement: Supplementary Table 4 [file bjc2017463x6.docx]

**Supplementary Table 4**. Sensitivity analyses for female models

|  | | **Colditz** | **Freedman** | **Guesmi** | | **Johnson** | | **QCancer10** | | **Tao** | | **Wei** | | **Wells** | **Driver*** | | **Ma (simple)*** | | **Ma (cox)*** |
| --- | --- | --- | --- | --- | --- | --- | --- | --- | --- | --- | --- | --- | --- | --- | --- | --- | --- | --- | --- |
|  | |  |  |  | |  | |  | |  | |  | |  |  | |  | |  |
| 5 year closed cohort | | |  |  | |  | |  | |  | |  | |  |  | |  | |  |
| n | | *n* = 164,034 | *n* = 130,188 | *n* = 202,620 | | *n* = 203,390 | | *n* = 193,365 | | *n* = 189,097 | | *n* = 194,601 | | *n* = 191,475 | *n* = 201,474 | | *n* = 174,297 | | *n* = 174,297 |
| CRC cases | | *n* = 592 | *n* = 562 | *n* = 752 | | *n* = 754 | | *n* = 714 | | *n* = 696 | | *n* = 716 | | *n* = 713 | *n* = 745 | | *n* = 628 | | *n* = 628 |
| AUC  (95% CI) | | 0.50  (0.48-0.53) | 0.59  (0.56-0.61) | 0.63  (0.61-0.64) | | 0.50  (0.48-0.52) | | 0.66  (0.64-0.67) | | 0.63  (0.61-0.65) | | 0.49  (0.47-0.51) | | 0.64  (0.62-0.66) | 0.63  (0.61-0.65) | | 0.64  (0.62-0.66) | | 0.64  (0.62-0.66) |
|  | |  |  |  | |  | |  | |  | |  | |  |  | |  | |  |
| Physical activity missing data | | |  |  | |  | |  | |  | |  | |  |  | |  | |  |
|  | | Missing values set to 10^th^ centile for continuous variables and zero for categorical | | | | | | | | | | | | |  | |  | |  |
| n | | *n* = 182,805 | *n* = 137,982 | n/a | | *n* = 203,390 | | n/a | | n/a | | n/a | | n/a | n/a | | *n* = 199,281 | | *n* = 199,281 |
| CRC cases | | *n* = 676 | *n* = 611 | n/a | | *n* = 754 | | n/a | | n/a | | n/a | | n/a | n/a | | *n* = 739 | | *n* = 739 |
| AUC  (95%CI) | | 0.50  (0.48-0.52) | 0.59  (0.57-0.61) | n/a | | 0.48  (0.46-0.50) | | n/a | | n/a | | n/a | | n/a | n/a | | 0.64  (0.62-0.65) | | 0.63  (0.62-0.65) |
|  | | Missing values set to 90^th^ centile for continuous variables and present for categorical | | | | | | | | | | | | |  | |  | |  |
| n | | *n* = 182,805 | *n* = 137,982 | n/a | | *n* = 203,390 | | n/a | | n/a | | n/a | | n/a | n/a | | *n* = 199,218 | | *n* = 199,218 |
| CRC cases | | *n* = 676 | *n* = 611 | n/a | | *n* = 754 | | n/a | | n/a | | n/a | | n/a | n/a | | *n* = 739 | | *n* = 739 |
| AUC  (95% CI) | | 0.51  (0.48-0.53) | 0.59  (0.56-0.61) | n/a | | 0.48  (0.46-0.50) | | n/a | | n/a | | n/a | | n/a | n/a | | 0.63  (0.61-0.65) | | 0.64  (0.62-0.65) |
|  | | |  |  | |  | |  | |  | |  | |  |  | |  | |  |
| NSAIDs/aspirin – removing NSAIDs/aspirin variables from risk models | | | | | | | | | |  | |  | |  |  | |  | |  |
| n | | *n* = 165,119 | *n* = 131,052 | n/a | | *n* = 203,390 | | n/a | | *n* = 190,553 | | n/a | | *n* = 192,925 | n/a | | n/a | | n/a |
| CRC cases | | *n* = 597 | *n* = 616 | n/a | | *n* = 754 | | n/a | | *n* = 702 | | n/a | | *n* = 718 | n/a | | n/a | | n/a |
| AUC  (95% CI) | | 0.51  (0.48-0.53) | 0.59  (0.57-0.61) | n/a | | 0.50  (0.48-0.52) | | n/a | | 0.64  (0.62-0.66) | | n/a | | 0.64  (0.63-0.67) | n/a | | n/a | | n/a |
|  | |  |  |  | |  | |  | |  | |  | |  |  | |  | |  |
| Hormonal factors – removing hormonal variables for risk models | | | | | | | | | | | |  | |  |  | |  | |  |
| n | | *n* = 164,807 | *n* = 130,790 | n/a | | *n* = 203,390 | | n/a | | n/a | | n/a | | *n* =192,603 | n/a | | n/a | | n/a |
| CRC cases | | *n* = 593 | *n* = 618 | n/a | | *n* = 754 | | n/a | | n/a | | n/a | | *n* =716 | n/a | | n/a | | n/a |
| AUC  (95% CI) | | 0.51  (0.48-0.53) | 0.59  (0.57-0.62) | n/a | | 0.50  (0.47-0.52) | | n/a | | n/a | | n/a | | 0.64  (0.62-0.66) | n/a | | n/a | | n/a |
|  | |  |  |  | |  | |  | |  | |  | |  |  | |  | |  |
| Excluding those with history of colorectal adenoma or inflammatory bowel disease | | | | | | | | | | | | | | |  | |  | |  |
| n | | *n* = 162,307 | *n* = 128,715 | *n* = 200,353 | | *n* = 201,115 | | *n* = 191,223 | | *n* = 187,010 | | *n* = 192,433 | | *n* = 189,366 | *n* = 199,222 | | *n* = 172,409 | | *n* = 172,409 |
| CRC cases | | *n* = 582 | *n* = 551 | *n* = 741 | | *n* = 743 | | *n* = 703 | | *n* = 685 | | *n* = 705 | | *n* = 702 | *n* = 734 | | *n* = 618 | | *n* = 618 |
| AUC  (95% CI) | | 0.50  (0.48-0.53) | 0.59  (0.57-0.61) | 0.63  (0.61-0.64) | | 0.50  (0.47-0.52) | | 0.66  (0.64-0.68) | | 0.64  (0.62-0.66) | | 0.49  (0.47-0.51) | | 0.64  (0.62-0.66) | 0.63  (0.61-0.65) | | 0.63  (0.61-0.65) | | 0.64  (0.62-0.66) |
|  |  | |  |  |  | |  | |  | |  | |  | |  |  | |  | |
| Open cohort | | |  |  |  | |  | |  | |  | |  | |  |  | |  | |
| n | | *n* = 220,094 | *n =*173,122 | *n* = 271,213 | *n* = 272,445 | | *n* = 258,875 | | *n* = 253,094 | | *n* = 260,230 | | *n* = 255,946 | | *n* = 269,464 | *n* = 234,003 | | *n* = 234,003 | |
| CRC cases | | *n* = 984 | *n* = 944 | *n* = 1,249 | *n* = 1,255 | | *n* = 1,189 | | *n* = 1,166 | | *n* = 1,193 | | *n* = 1,185 | | *n* = 1,240 | *n* = 1,045 | | *n* = 1,045 | |
| C-statistic  (95% CI) | | 0.52  (0.50-0.54) | 0.59  (0.57-0.62) | 0.63  (0.62-0.65) | 0.50  (0.48-0.52) | | 0.65  (0.64-0.67) | | 0.64  (0.62-0.65) | | 0.50  (0.48-0.52) | | 0.65  (0.64-0.67) | | 0.63  (0.61-0.65) | 0.64  (063-0.66) | | 0.64  (0.62-0.66) | |

AUC = area under the receiver operating characteristic curve * Models originally developed only for men.
