# Supplementary material for: External validation of risk prediction models for incident colorectal cancer using UK Biobank
Source: Br J Cancer. 2018 Jan 30;118(5):750–9. doi: 10.1038/bjc.2017.463 (PMC5846069; doi:10.1038/bjc.2017.463)
Supplement: Supplementary Table 5 [file bjc2017463x7.docx]

**Supplementary Table 5**. Characteristics of the open cohort

|  | **No incident CRC**  ***n* = 497,354** | **Incident CRC**  ***n* = 2,945** | **% with incident CRC** |
| --- | --- | --- | --- |
| Age (years) |  |  |  |
| Mean (SD) | 56.48 (8.10) | 61.15 (6.43) | --- |
| Missing (%) | 0 (0) | 0 (0) | --- |
| Sex |  |  |  |
| Male (%) | 226,164 (45.47) | 1,690 (57.39) | 0.74 |
| Female (%) | 271,190 (54.53) | 1,255 (42.61) | 0.46 |
| Missing (%) | 0 (0) | 0 (0) | --- |
| Ethnicity |  |  |  |
| White (%) | 467,726 (94.04) | 2,839 (96.99) | 0.61 |
| Other (%) | 26,876 (5.40) | 88 (3.01) | 0.33 |
| Missing (%) | 2,752 (0.55) | 18 (0.61) | 0.65 |
| Years of full time education |  |  |  |
| Mean (SD) | 13.11 (2.80) | 12.76 (2.86) | --- |
| Missing (%) | 10,585 (2.13) | 65 (2.21) | --- |
| BMI (kg/m^2^) |  |  |  |
| <20 (%) | 11,640 (2.34) | 43 (1.47) | 0.37 |
| 20-24.9 (%) | 151,987 (30.56) | 749 (25.61) | 0.49 |
| 25-29.9 (%) | 209,773 (42.18) | 1,327 (45.37) | 0.63 |
| ≥30 (%) | 120,884 (24.31) | 806 (27.56) | 0.67 |
| Missing (%) | 3,070 (0.62) | 20 (0.68) | 0.65 |
| Family history of CRC* |  |  |  |
| Yes (%) | 53,307 (10.72) | 430 (14.60) | 0.81 |
| No (%) | 424,196 (85.29) | 2,395 (81.32) | 0.56 |
| Missing (%) | 19,851 (3.99) | 120 (4.07) | 0.60 |
| Smoking status |  |  |  |
| Never (%) | 271,192 (54.53) | 1,344 (46.01) | 0.50 |
| Former (%) | 170,740 (34.33) | 1,284 (43.96) | 0.75 |
| Current (%) | 52,516 (10.56) | 293 (10.03) | 0.56 |
| Missing (%) | 2,906 (0.58) | 24 (0.81) | 0.83 |
| Alcohol drinking status |  |  |  |
| Non (%) | 22,331 (4.49) | 109 (3.71) | 0.49 |
| Former (%) | 17,894 (3.60) | 110 (3.75) | 0.61 |
| Current (%) | 455,641 (91.61) | 2,716 (92.54) | 0.60 |
| Missing (%) | 1,488 (0.30) | 10 (0.34) | 0.67 |
| Physical activity (MET-h/d) |  |  |  |
| Mean (SD) | 28.25 (5.01) | 28.56 (5.60) | --- |
| Missing (%) | 57,165 (11.49) | 397 (13.48) | --- |
| Red meat consumption |  |  |  |
| <3 times/week (%) | 380,838 (76.57) | 2,161 (73.4) | 0.57 |
| ≥3 times/week (%) | 109,573 (22.03) | 748 (25.4) | 0.68 |
| Missing (%) | 6,943 (1.40) | 36 (1.22) | 0.52 |
| Current aspirin use |  |  |  |
| Yes (%) | 71,380 (14.35) | 613 (21.05) | 0.86 |
| No (%) | 420,233 (83.49) | 2,299 (78.95) | 0.54 |
| Missing (%) | 5,741 (1.15) | 33 (1.12) | 0.57 |
| Current NSAID use |  |  |  |
| Yes (%) | 152,272 (30.62) | 979 (33.58) | 0.64 |
| No (%) | 339,823 (68.33) | 1,936 (66.42) | 0.57 |
| Missing (%) | 5,259 (1.06) | 30 (1.02) | 0.57 |
| Fruit and vegetable consumption |  |  |  |
| <5 portions/day (%) | 110,678 (22.25) | 638 (21.7) | 0.58 |
| ≥5 portions/day (%) | 375,073 (75.41) | 2,232 (75.8) | 0.60 |
| Missing (%) | 11,603 (2.33) | 75 (2.55) | 0.64 |

CRC – colorectal cancer; BMI – body mass index; MET-h/d – Metabolic Equivalent of Task hours per day; NSAID – non steroidal anti-inflammatory drug

* One of more of mother, father or sibling
