# Supplementary material for: Correction: External validation of risk prediction models for incident colorectal cancer using UK Biobank
Source: Br J Cancer. 2020 Mar 12;122(10):1572–5. doi: 10.1038/s41416-020-0767-0 (PMC7217758; doi:10.1038/s41416-020-0767-0)
Supplement: Supplementary file 1 — Supplemental Material [file 41416_2020_767_MOESM1_ESM.docx]

**Supplementary Table 3**. Sensitivity analyses for male models

|  | | | **Colditz** | **Driver** | **Freedman** | **Guesmi** | **Johnson** | **Ma (simple)** | **Ma (Cox)** | **QCancer10** | **Tao** | **Wei** | **Wells** |
| --- | --- | --- | --- | --- | --- | --- | --- | --- | --- | --- | --- | --- | --- |
|  |  | | | | | | | | | | | | |
| 5 year closed cohort | | | | | | | | | | | | | |
| n | | | *n* = 139,257 | *n* = 167,762 | *n =*101,530 | *n* = 168,825 | *n* = 169,722 | *n* = 150,386 | *n* = 150,386 | *n* = 158,024 | *n* = 149,693 | *n* = 160,256 | *n* = 140,917 |
| CRC cases | | | *n* =761 | *n* = 946 | *n* = 685 | *n* = 961 | *n* = 965 | *n* = 830 | *n* = 830 | *n* = 884 | *n* = 825 | *n* = 898 | *n* = 764 |
| AUC  (95% CI) | | | 0.56  (0.54-0.58) | 0.67  (0.66-0.69) | 0.64  (0.61-0.66) | 0.65  (0.63-0.66) | 0.49  (0.47-0.51) | 0.68  (0.67-0.70) | 0.69  (0.68-0.71) | 0.70  (0.69-0.72) | 0.69  (0.67-0.70) | 0.51  (0.49-0.53) | 0.71  (0.69-0.72) |
|  | | |  |  |  |  |  |  |  |  |  |  |  |
| Physical activity missing data | | | |  |  |  |  |  |  |  |  |  |  |
|  | Missing values set to 10^th^ centile for continuous variables and zero for categorical | | | | | | | | | | | | |
| n | | | *n* = 148,957 | n/a | *n =*108,306 | n/a | *n* = 169,722 | *n* = 165,347 | *n* = 165,347 | n/a | n/a | n/a | *n* = 152,565 |
| CRC cases | | | *n* = 828 | n/a | *n* = 746 | n/a | *n* = 965 | *n* = 933 | *n* = 933 | n/a | n/a | n/a | *n* = 849 |
| AUC  (95%CI) | | | 0.55  (0.53-0.57) | n/a | 0.64  (0.62-0.66) | n/a | 0.48  (0.46-0.50) | 0.68  (0.67-0.70) | 0.69  (0.68-0.71) | n/a | n/a | n/a | 0.71  (0.69-0.72) |
|  | Missing values set to 90^th^ centile for continuous variables and present for categorical | | | | | | | | | | | | |
| n | | | *n* = 148,957 | n/a | *n =*108,306 | n/a | *n* = 169,722 | *n* = 165,347 | *n* = 165,347 | n/a | n/a | n/a | *n* = 152,565 |
| CRC cases | | | *n* = 828 | n/a | *n* = 746 | n/a | *n* = 965 | *n* = 933 | *n* = 933 | n/a | n/a | n/a | *n* = 849 |
| AUC  (95% CI) | | | 0.56  (0.54-0.58) | n/a | 0.64  (0.62-0.66) | n/a | 0.48  (0.46-0.50) | 0.68  (0.66-0.69) | 0.69  (0.68-0.71) | n/a | n/a | n/a | 0.71  (0.69-0.72) |
|  | | | |  |  |  |  |  |  |  |  |  |  |
| NSAIDs/aspirin – removing NSAIDs/aspirin variables from risk models | | | | | | | | | |  |  |  |  |
| n | | *n* = 140,437 | | n/a | *n* =102,405 | n/a | *n* = 169,722 | n/a | n/a | n/a | *n* = 151,082 | n/a | *n* = 142,187 |
| CRC cases | | *n* = 771 | | n/a | *n* = 759 | n/a | *n* = 965 | n/a | n/a | n/a | *n* = 837 | n/a | *n* = 774 |
| AUC  (95% CI) | | 0.57  (0.55-0.59) | | n/a | 0.64  (0.62-0.66) | n/a | 0.49  (0.47-0.51) | n/a | n/a | n/a | 0.69  (0.67-0.71) | n/a | 0.71  (0.69-0.72) |
|  | |  | |  |  |  |  |  |  |  |  |  |  |
| Deprivation and years of education – removing deprivation from risk models | | | | | | | | | | | | | |
| n | | n/a | | n/a | n/a | n/a | n/a | n/a | n/a | *n* = 158,222 | n/a | n/a | n/a |
| CRC cases | | n/a | | n/a | n/a | n/a | n/a | n/a | n/a | *n* = 885 | n/a | n/a | n/a |
| AUC  (95% CI) | | n/a | | n/a | n/a | n/a | n/a | n/a | n/a | 0.70  (0.69-0.72) | n/a | n/a | n/a |
|  | |  | |  |  |  |  |  |  |  |  |  |  |
| Excluding those with history of colorectal adenoma or inflammatory bowel disease at baseline | | | | | | | | | | | | | |
| n | | *n* =137,676 | | *n* = 165,847 | *n* = 100,329 | *n* = 166,895 | *n* = 167,790 | *n* = 148,671 | *n* = 148,671 | *n* = 156,209 | *n* = 147,980 | *n* = 158,415 | *n* =139,316 |
| CRC cases | | *n* =747 | | *n* = 928 | *n* = 671 | *n* = 942 | *n* = 946 | *n* = 816 | *n* = 816 | *n* = 867 | *n* = 808 | *n* = 880 | *n* = 751 |
| AUC  (95% CI) | | 0.56  (0.54-0.58) | | 0.68  (0.66-0.69) | 0.64  (0.61-0.66) | 0.65  (0.63-0.66) | 0.49  (0.47-0.51) | 0.68  (0.67-0.70) | 0.70  (0.68-0.71) | 0.70  (0.69-0.72) | 0.69  (0.67-0.71) | 0.51  (0.49-0.53) | 0.71  (0.69-0.73) |
|  | |  | |  |  |  |  |  |  |  |  |  |  |
| Open cohort | | | |  |  |  |  |  |  |  |  |  |  |
| n | | | *n* = 187,221 | *n* = 224,810 | *n =*135,802 | *n* = 226,438 | *n* = 227,854 | *n* = 202,095 | *n* = 202,095 | *n* = 211,747 | *n* = 200,937 | *n* = 214,677 | *n* = 189,356 |
| CRC cases | | | *n* = 1,347 | *n* = 1,661 | *n* = 1,203 | *n* = 1,683 | *n* = 1,690 | *n* = 1,457 | *n* = 1,457 | *n* = 1,559 | *n* = 1,454 | *n* = 1,589 | *n* = 1,340 |
| C-statistic  (95% CI) | | | 0.53  (0.52-0.55) | 0.68  (0.67-0.69) | 0.64  (0.62-0.66) | 0.66  (0.64-0.67) | 0.47  (0.46-0.49) | 0.68  (0.67-0.70) | 0.69  (0.68-0.71) | 0.70  (0.69-0.72) | 0.69  (0.67-0.70) | 0.50  (0.49-0.52) | 0.71  (0.69-0.73) |

AUC = area under the receiver operating characteristic curve
